# *In Silico* Exploration of Cannabidiol Interactions with Outer Membrane Proteins in *Salmonella* Typhimurium LT2

**DOI:** 10.3390/ijms27188247

**Published:** 2026-09-16

**Authors:** Iddrisu Ibrahim, Emmanuel Ndezure, Kelci Lawrence, Othreniel Angel Forte, Navitri Naidu, Latrell Huitt, Nathaniel Ajibola, Junhuan Xu, Robertson K. Boakai, Moses Mengu, Fortune Akabanda, James Owusu-Kwarteng, Olufemi S. Ajayi, Joseph Atia Ayariga

**Affiliations:** 1 The Microbiology Program, College of Science, Technology, Engineering, and Mathematics (C-STEM), Alabama State University, Montgomery, AL 36104, USA; iiddrisu3715@alasu.edu (I.I.); endezure@alasu.edu (E.N.); klawrence0670@alasu.edu (K.L.); oforte3773@myasu.alasu.edu (O.A.F.); nnaidu@alasu.edu (N.N.); lhuitt@alasu.edu (L.H.); najibola7327@alasu.edu (N.A.); jxu@alasu.edu (J.X.); brobertson@alasu.edu (R.K.B.); 2 The Industrial Hemp Program, College of Science, Technology, Engineering, and Mathematics (C-STEM), Alabama State University, Montgomery, AL 36104, USA; 3 African Drug Discovery and Innovation Network, Sunyani P.O. Box AB45, Ghana; mosesmengu@gmail.com (M.M.); james.owusu-kwarteng@uenr.edu.gh (J.O.-K.); 4 Counsil for Scientific and Industrial Research (CSIR), College of Science and Technology (CCST), Josip Broz Tito Avenue (GL-063-0751), Accra P.O. Box M32, Ghana; 5 Department of Food Science and Technology, University for Development Studies, Tamale P.O. Box TL1350, Ghana; fakabanda@uds.edu.gh; 6 Department of Food Science and Technology, School of Agriculture and Technology, University of Energy and Natural Resources, Sunyani P.O. Box 214, Ghana

**Keywords:** outer membrane proteins, Gram-negative bacteria, antibiotic resistance, cannabidiol, phylogenetics, in silico

## Abstract

Cannabidiol (CBD), the non-psychoactive component of the hemp plant, has been investigated as a potential novel antimicrobial agent. This study was aimed at understanding the interactions between CBD and the outer membrane proteins (OMPs) of *Salmonella* Typhimurium LT2. Employing *in silico* techniques, we analyzed the binding affinities, interaction profiles, and drug-likeness of CBD with five independent OMP targets—OmpA, OmpC, OmpD, OmpF, and OmpX—together with a historical OmpD/nmpC receptor entry (legacy NmpC label). The molecular docking results showed varying predicted binding affinities across the evaluated OMP receptor entries, with OmpX exhibiting a docking score of −6.6 kcal/mol and the OmpD/nmpC legacy receptor entry, originally labeled “NmpC,” exhibiting a score of −6.4 kcal/mol. The latter was retained for transparency but was not interpreted as an independent OMP target following annotation verification. The high sequence conservation observed among the analyzed OMPs further suggests that structurally similar regions may occur across multiple *Salmonella* strains. KEGG pathway mapping was used to provide functional context for OmpC and OmpF, including their association with two-component signaling and β-lactam-resistance pathways. These pathway analyses contextualize the biological roles of the porins but do not demonstrate that CBD directly alters the mapped pathways. Similarly, the docking analyses do not establish functional inhibition of these proteins or demonstrate effects on membrane integrity, bacterial survival, virulence, or antimicrobial susceptibility. Rather, the findings identify candidate CBD–OMP interactions and mechanistic hypotheses that warrant experimental validation. In conclusion, the in silico findings provide structural evidence of predicted interactions between CBD and selected OMPs of *Salmonella* Typhimurium LT2 and lay the foundation for further experimental studies to determine their biological significance.

## 1. Introduction

*Salmonella* Typhimurium is an important foodborne pathogen that causes infections worldwide. Its outer membrane proteins (OMPs) contribute to envelope integrity, nutrient acquisition, environmental survival, and interactions with the host [1,2]. Porins such as OmpC and OmpF permit the passive diffusion of small hydrophilic molecules and can support bacterial growth in nutrient-limited environments, including the host gut [3].

Furthermore, the Gram-negative outer membrane is an asymmetric permeability barrier that protects *Salmonella* Typhimurium against environmental stress and antimicrobial agents [4]. In addition, OmpA and OmpD support envelope stability through interactions with the peptidoglycan layer [5]. Other OMPs contribute to host interactions: OmpX has been associated with immune evasion, whereas OmpA can support adhesion and invasion [6,7,8,9]. Cannabidiol (CBD) is a non-intoxicating phytocannabinoid that has attracted considerable therapeutic interest, including investigation of its antimicrobial properties. The antibacterial activity of cannabinoids has been recognized for several decades, with early quantitative studies demonstrating inhibitory activity of purified CBD against bacterial species. More recent studies have confirmed antibacterial activity of CBD against several clinically relevant bacteria and have provided evidence that membrane disruption and altered membrane permeability may contribute to its antibacterial effects [10,11,12,13]. CBD has also demonstrated activity in combination with selected conventional antibiotics, including in our previous studies with *Salmonella* Typhimurium [10,11,12,13]. These properties suggest that CBD may offer potential value as an antimicrobial lead or antibiotic adjuvant, particularly because its membrane-associated activity differs from the primary targets of many conventional antibiotics. However, CBD is not currently a replacement for established antibacterial therapy, and further mechanistic and experimental validation is required. In our quest to explore CBD’s potential as an antimicrobial agent, we examined its predicted interactions with outer membrane proteins (OMPs) of *Salmonella* Typhimurium. We hypothesized that CBD may interact with these proteins at structurally relevant binding sites and that such interactions could provide testable hypotheses regarding possible effects on OMP-associated processes, including nutrient transport, membrane organization, and host interactions. Previous experimental studies, including work from our group, have demonstrated the antibacterial activity of CBD against *Salmonella* and other bacterial pathogens using minimum inhibitory concentration (MIC) and related susceptibility assays [10,11,12,13]. Therefore, the objective of the present study was to investigate whether CBD could interact with selected outer membrane proteins of *Salmonella* Typhimurium LT2 as potential molecular interaction partners associated with membrane-related antibacterial effects. The present docking analysis therefore represents a mechanistic exploration of a previously demonstrated antibacterial phenotype rather than an independent experimental validation of CBD antimicrobial activity.

To achieve this, we used computational studies to determine the predicted binding affinities of CBD to five independent *Salmonella* Typhimurium OMP targets—OmpA, OmpC, OmpD, OmpF, and OmpX—together with the historical OmpD/nmpC receptor entry (legacy NmpC label). We identified and characterized the binding sites and residues involved in the predicted interactions between CBD and the OMPs. We also examined root-mean-square deviation (RMSD) values to assess the geometric variation in alternative docking poses relative to the top-ranked pose [14]. We further explored the genetic diversity and evolutionary relationships of the OMPs among varied *Salmonella enterica* strains using phylogenetic analysis. Finally, we analyzed the drug-likeness of CBD using ADMET predictions and examined the pathways associated with OmpC and OmpF, given their roles in nutrient transport and permeability. Thus, this study provides a deeper understanding of the predicted interactions between CBD and *Salmonella* OMPs and establishes a basis for further experimental investigation of their biological significance.

## 2. Results

**Figure 1:**
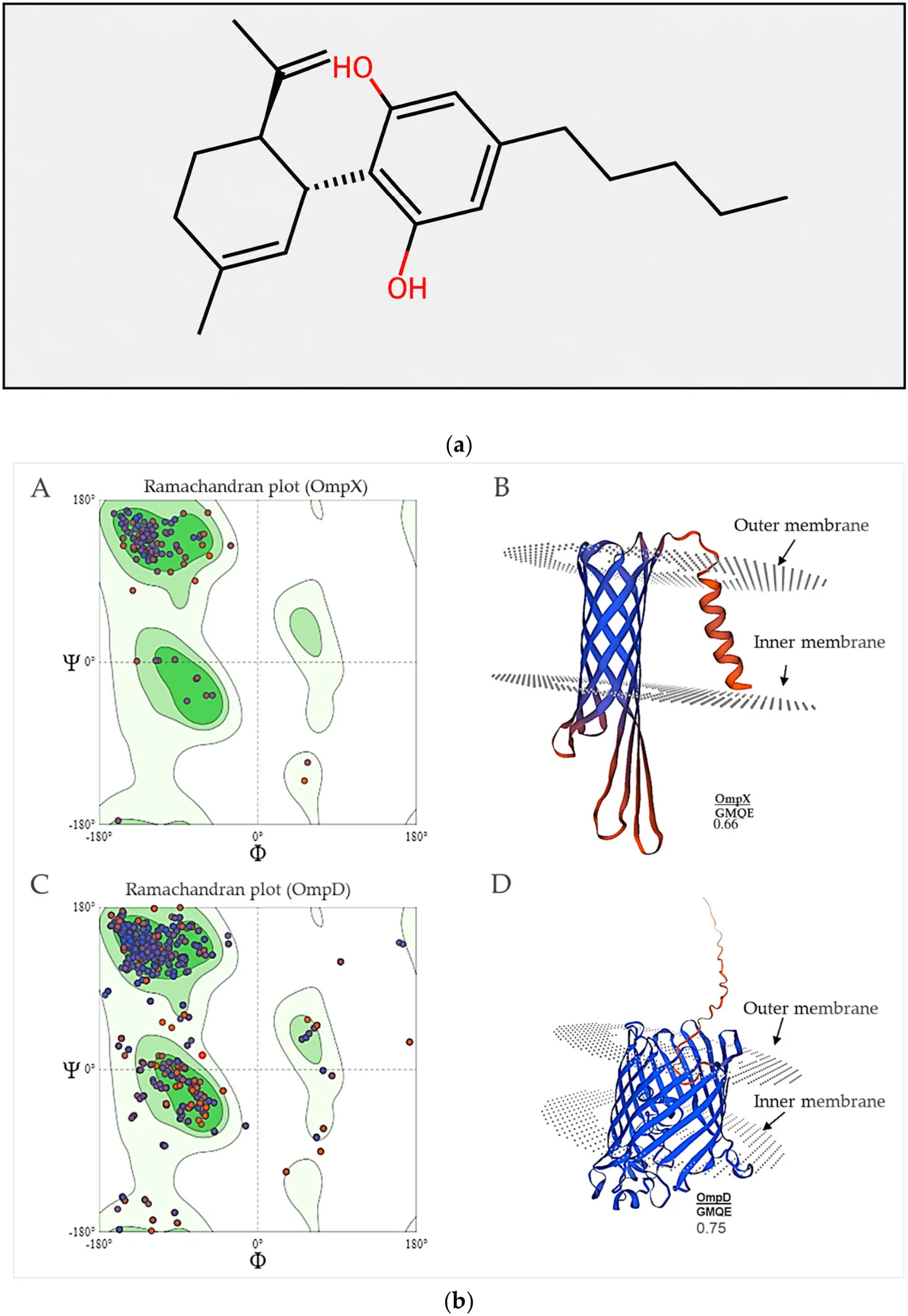
(**a**) CBD 2D structure from PubChem [https://pubchem.ncbi.nlm.nih.gov/compound/644019#section=2D-Structure (accessed on 1 September 2023)]. (**b**) Ramachandran plots (**A**,**C**) and 3D models of OmpX (**B**) and OmpD (**D**).

Figure 1a presents the 2D chemical structure of cannabidiol (CBD), obtained from PubChem. The structure shows the chemical configuration of CBD, including its hydroxyl groups, aromatic rings, and hydrocarbon moieties relevant to subsequent molecular docking analysis.

Following characterization of the CBD ligand, the receptor structures used for molecular docking were prepared. Experimentally available OMP structures were retrieved from the RCSB Protein Data Bank, whereas OmpX and OmpD required homology modeling using SWISS-MODEL. Therefore, OmpX and OmpD are specifically presented in Figure 1b together with their Ramachandran plots and model-quality parameters to document the structural quality of the modeled receptors before their use in the docking analyses.

The Ramachandran plots for OmpX and OmpD are illustrated in Figure 1b(A,C) and show the distribution of the phi (Φ) and psi (Ψ) torsion angles of the amino acid residues in the two modeled OMPs. The plots show favored regions associated with different secondary-structure conformations. The green-shaded areas represent regions in which β-sheet conformations are favored. Data points within these regions indicate that residues in OmpX and OmpD adopt β-sheet conformations. Less densely populated regions may represent residues adopting other conformations, including α-helical regions. Figure 1b(B,D) presents the three-dimensional structural models of OmpX and OmpD. Both proteins display transmembrane β-barrel structural features characteristic of outer membrane proteins. The reported GMQE scores were 0.66 for OmpX and 0.75 for OmpD.

**Figure 2:**
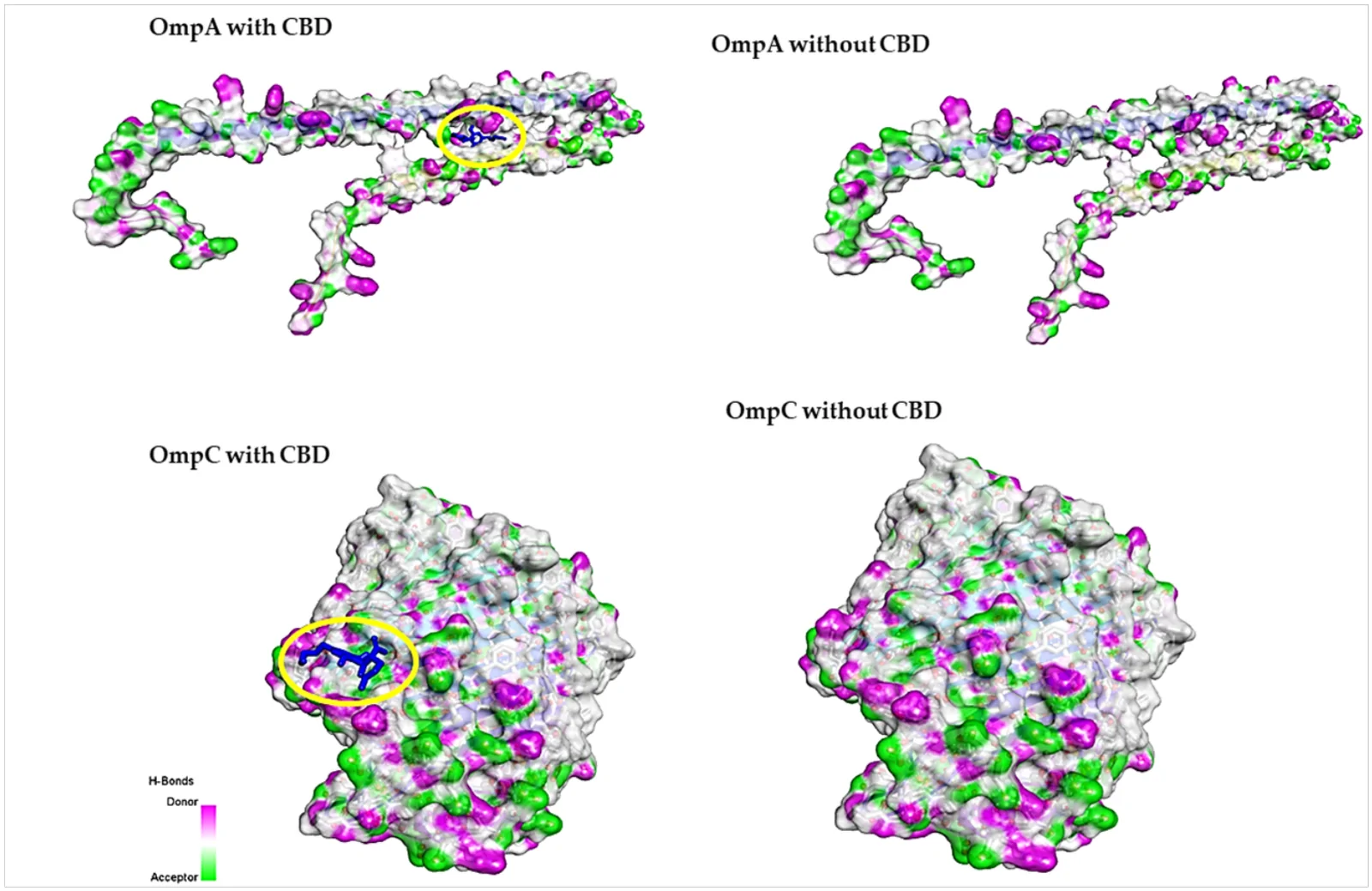
Surface representations of OmpA and OmpC in the unliganded state and corresponding docked CBD–protein complexes. CBD is shown in blue and circled in yellow. Protein structures were retrieved from the RCSB Protein Data Bank or modeled using SWISS-MODEL where required.

Figure 2 illustrates surface representations of OmpA and OmpC in their unliganded forms and corresponding docked CBD–protein complexes, with CBD highlighted and circled in yellow. The protein structural data were obtained from the RCSB Protein Data Bank and, where required, supplemented with structures generated using SWISS-MODEL. These representations were used to visualize the predicted location of CBD within the protein-binding regions. Although visual differences may be apparent between the displayed structures, no quantitative structural comparison was performed; therefore, these images are not interpreted as evidence of CBD-induced conformational changes.

**Figure 3:**
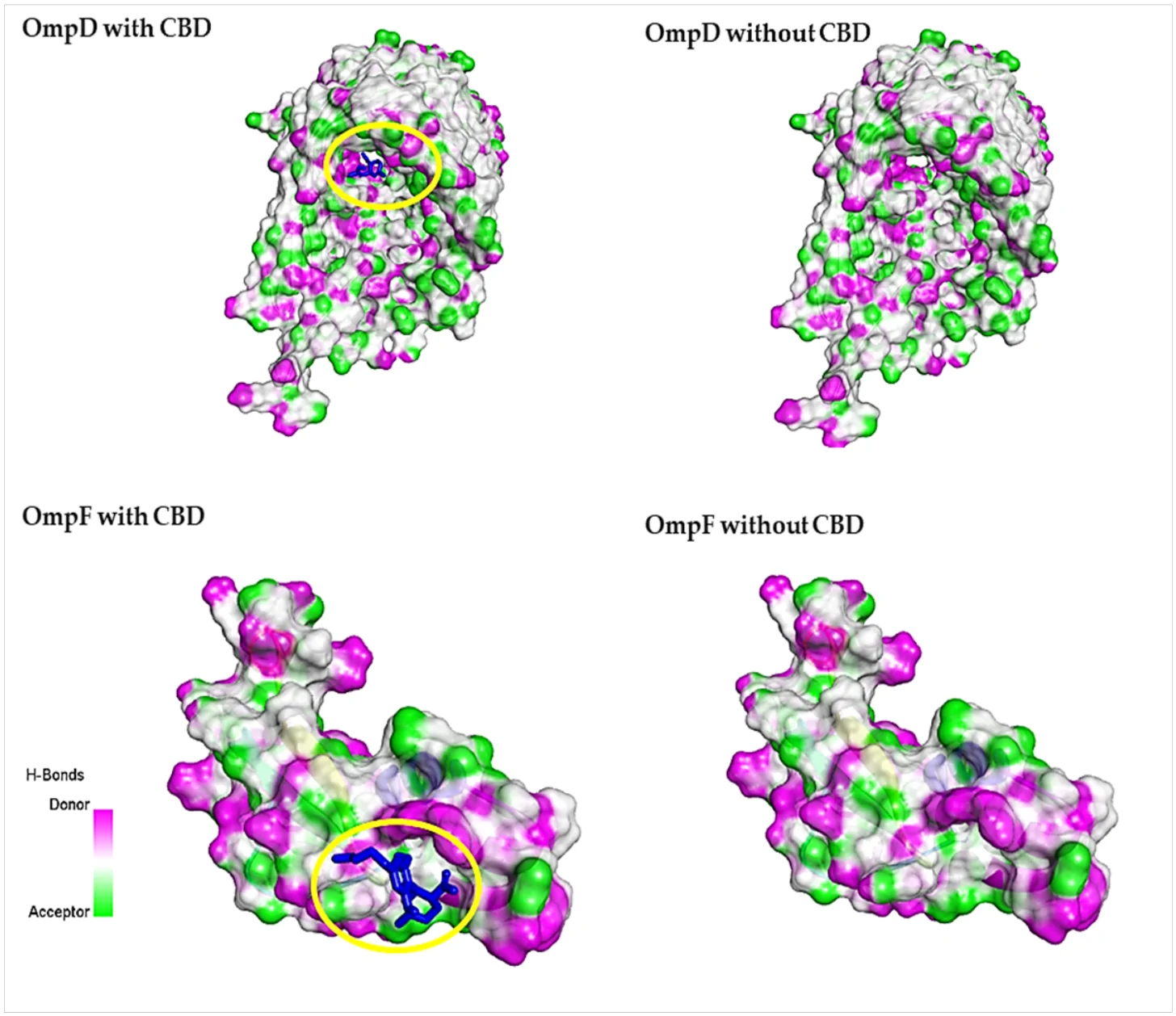
Surface representations of OmpD and OmpF in the unliganded state and corresponding docked CBD–protein complexes. CBD is shown in blue and circled in yellow. Protein structures were retrieved from the RCSB Protein Data Bank or modeled using SWISS-MODEL where required.

Similarly, Figure 3 presents surface representations of OmpD and OmpF in their unliganded forms and corresponding docked CBD–protein complexes. The structures were obtained from the RCSB Protein Data Bank or generated using SWISS-MODEL where required. The images illustrate the predicted positioning of CBD relative to the protein surfaces and binding regions. These visualizations were not subjected to quantitative structural superposition or conformational analysis and therefore are not interpreted as demonstrating CBD-induced changes in protein structure, permeability, or function.

**Figure 4:**
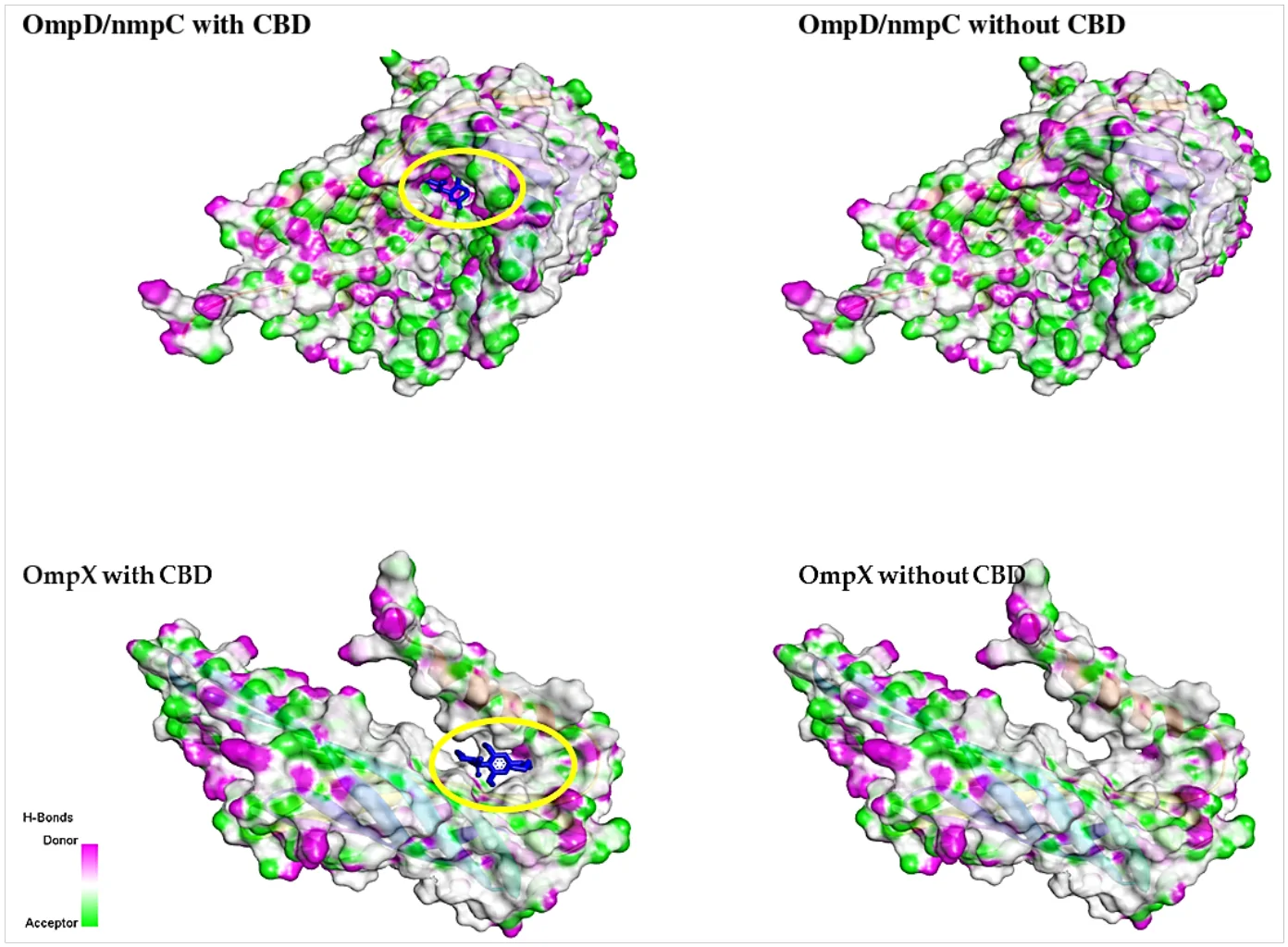
Surface representations of the OmpD/nmpC legacy receptor entry and OmpX in the unliganded state and corresponding docked CBD–protein complexes. CBD is shown in blue and circled in yellow.

Figure 4 presents surface representations of the OmpD/nmpC (legacy NmpC receptor entry) and OmpX in their unliganded forms and corresponding docked CBD–protein complexes. The visualizations identify the predicted locations of CBD within the respective protein-binding regions. They are intended to illustrate docking geometry and ligand positioning and are not interpreted as evidence of CBD-induced alterations in protein surface morphology or biological activity because no quantitative conformational comparison was performed.

**Figure 5:**
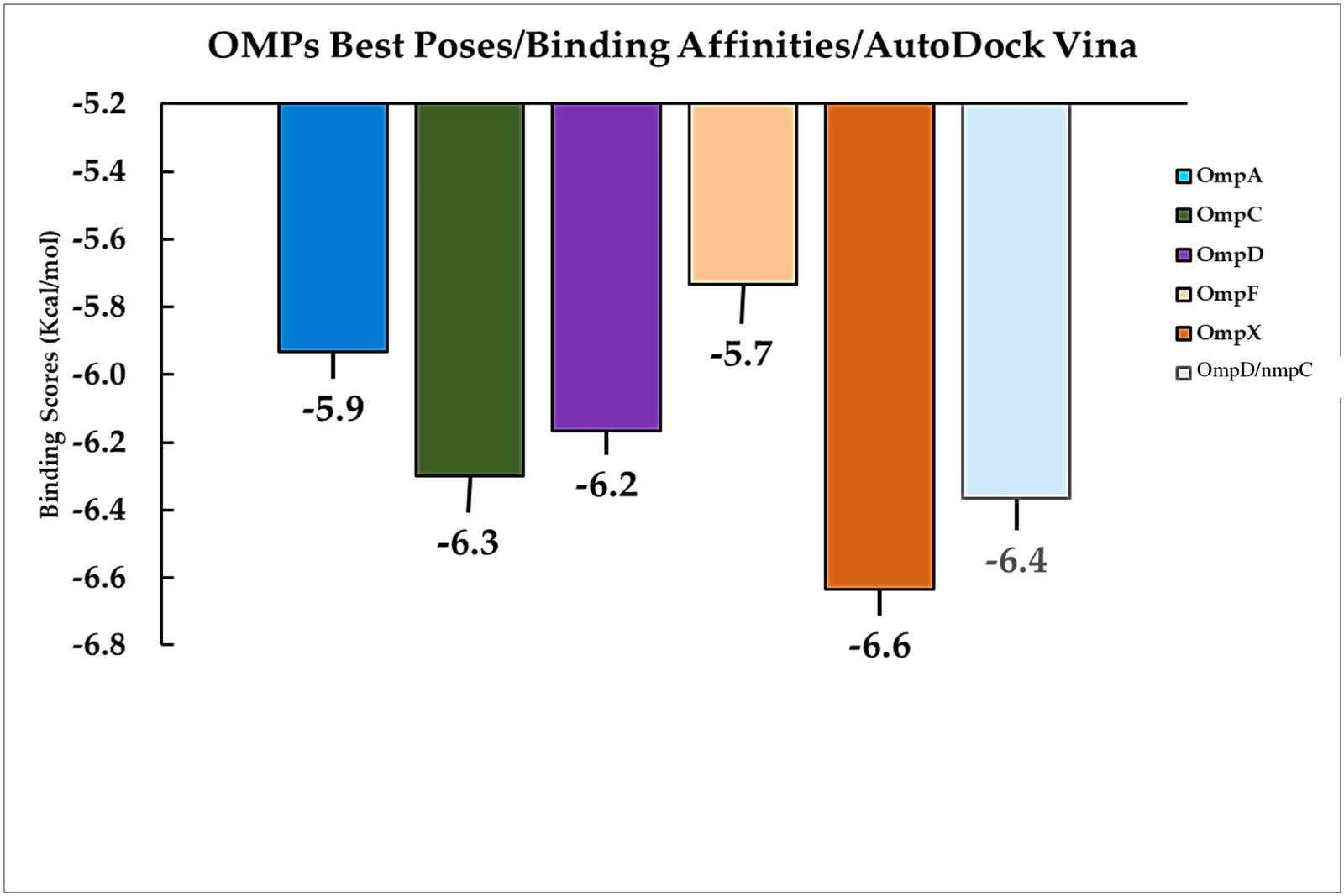
Predicted CBD-binding affinities for the evaluated *Salmonella* Typhimurium LT2 outer membrane protein receptor entries obtained using standalone AutoDock Vina. Values represent the mean ± SD of three independent docking runs (n = 3). Differences in mean binding affinities among receptor entries were assessed using one-way ANOVA (overall ANOVA, *p* = 0.005).

The predicted binding affinities of CBD with the different outer membrane protein (OMP) receptor entries from *Salmonella* Typhimurium LT2 are presented in Figure 5. For each receptor entry, the top-ranked pose from each of three independent docking runs was retained, and the corresponding binding scores were used to calculate the mean predicted binding affinity. The results showed that OmpX exhibited the most favorable predicted binding score at −6.6 kcal/mol. The receptor entry originally designated “NmpC” exhibited a predicted docking score of −6.4 kcal/mol. Subsequent annotation verification identified *nmpC* as a synonym of *ompD* in *Salmonella* Typhimurium LT2; therefore, this legacy receptor entry was retained in the reported docking results for transparency but was not considered an additional independent OMP target. OmpF exhibited the least favorable predicted binding score at −5.7 kcal/mol. Overall, the predicted binding scores varied among the evaluated receptor entries.

**Figure 6:**
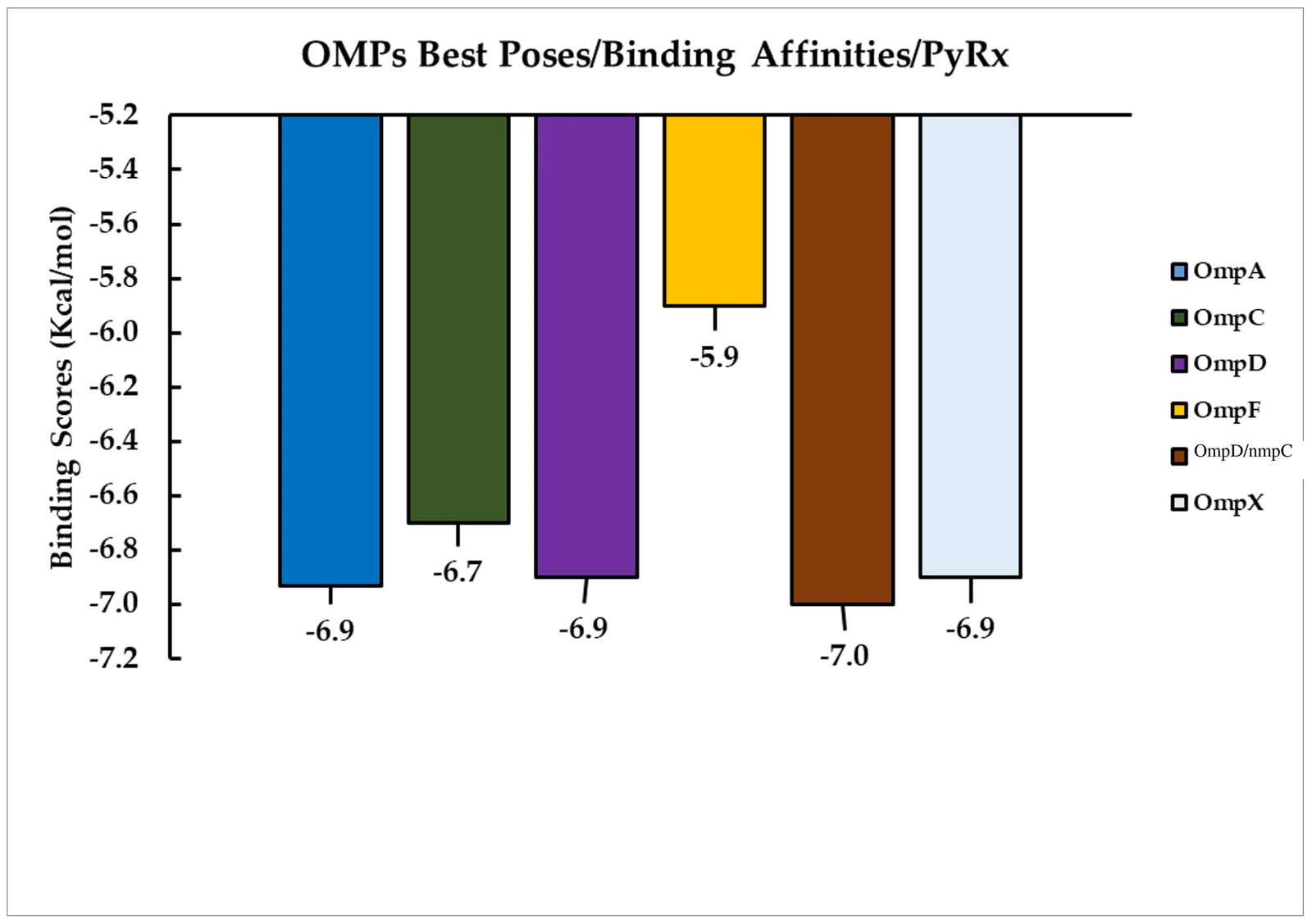
Predicted CBD-binding affinities for the evaluated *Salmonella* Typhimurium LT2 outer membrane protein receptor entries obtained using the PyRx–AutoDock Vina workflow. Values represent the mean ± SD of three independent docking runs (n = 3). Differences in mean binding affinities among receptor entries were assessed using one-way ANOVA (overall ANOVA, *p* = 0.005).

The PyRx–AutoDock Vina workflow produced predicted CBD-binding scores ranging from −5.9 to −7.0 kcal/mol across the evaluated receptor entries (Figure 6). The OmpD/nmpC legacy receptor entry produced the most favorable predicted score (−7.0 kcal/mol), whereas OmpF produced the least favorable score (−5.9 kcal/mol). OmpA, OmpD, and OmpX each produced a score of −6.9 kcal/mol, while OmpC produced a score of −6.7 kcal/mol.

**Figure 7:**
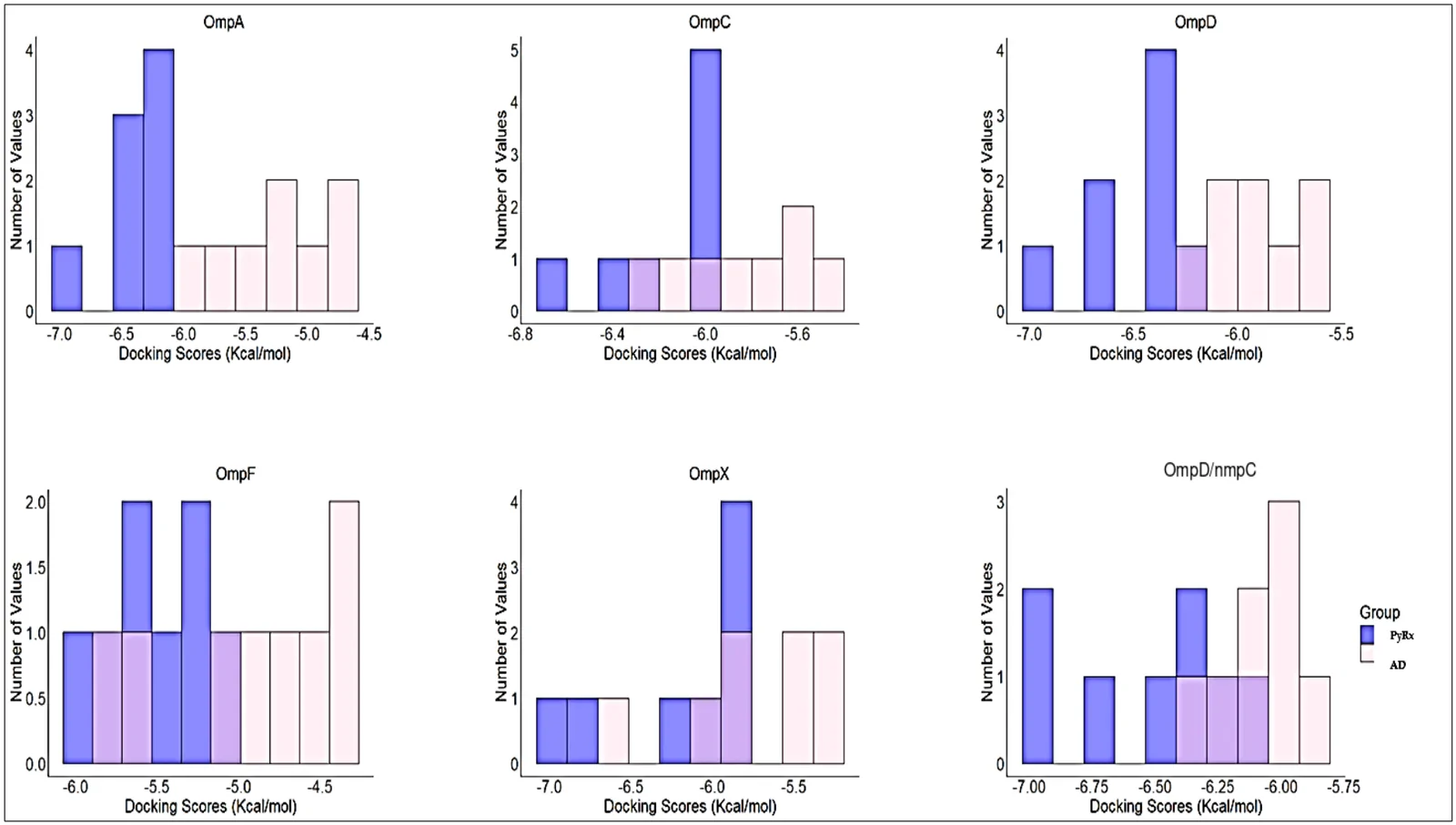
Comparison of predicted CBD-binding affinities obtained using the PyRx–AutoDock Vina workflow and standalone AutoDock Vina for the evaluated *Salmonella* Typhimurium LT2 outer membrane protein receptor entries. Values represent the mean ± SD of three independent docking runs per receptor and docking workflow (n = 3).

We included docking analysis carried out using standalone AutoDock Vina to compare the predicted binding scores with those obtained using the PyRx–AutoDock Vina workflow, as shown in Figure 7. The comparative analysis showed that the PyRx–AutoDock Vina workflow produced consistently more favorable predicted binding scores than standalone AutoDock Vina across the evaluated receptor entries. For instance, the OmpA predicted binding scores ranged from −6.5 to −6.0 kcal/mol using PyRx–AutoDock Vina and from −5.9 to −4.7 kcal/mol using standalone AutoDock Vina. Similar patterns were observed for the other evaluated receptor entries.

**Figure 8:**
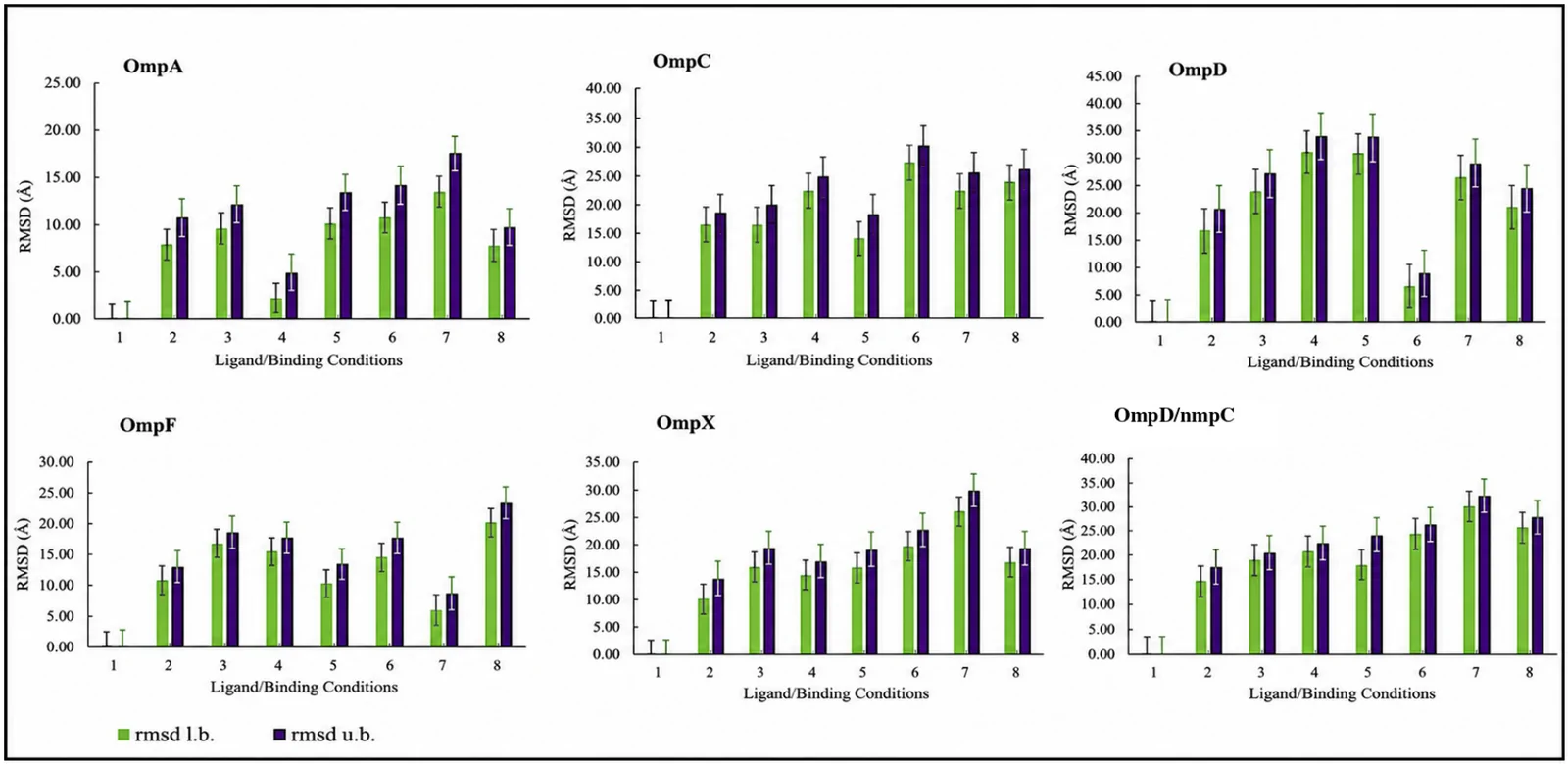
RMSD lower-bound (RMSD l.b.) and upper-bound (RMSD u.b.) values for alternative CBD docking poses relative to the top-ranked pose for the evaluated *Salmonella* Typhimurium LT2 outer membrane protein receptor entries. RMSD l.b. and RMSD u.b. describe the geometric divergence of alternative poses from the top-ranked reference pose and were not interpreted as measures of time-dependent binding stability.

As shown in Figure 8, the Root Mean Square Deviation (RMSD) of the predicted CBD–OMP docking poses was analyzed. Using AutoDock Vina, both RMSD lower-bound (RMSD l.b.) and upper-bound (RMSD u.b.) values were generated to describe the positional deviation in alternative docking poses relative to the top-ranked reference pose. Each OMP has an RMSD value of 0 Å for the top-ranked pose because this pose serves as the reference against which the remaining poses are compared. For subsequent poses, higher RMSD values indicate greater geometric divergence from the reference pose rather than changes in binding stability over time. The range of RMSD values therefore reflects variation in the predicted CBD-binding orientations obtained during docking. Consequently, these RMSD values were used to compare the geometric similarity of the different docking poses and were not interpreted as evidence of time-dependent stability, dynamic binding behavior, or sustained ligand–protein associations.

**Figure 9:**
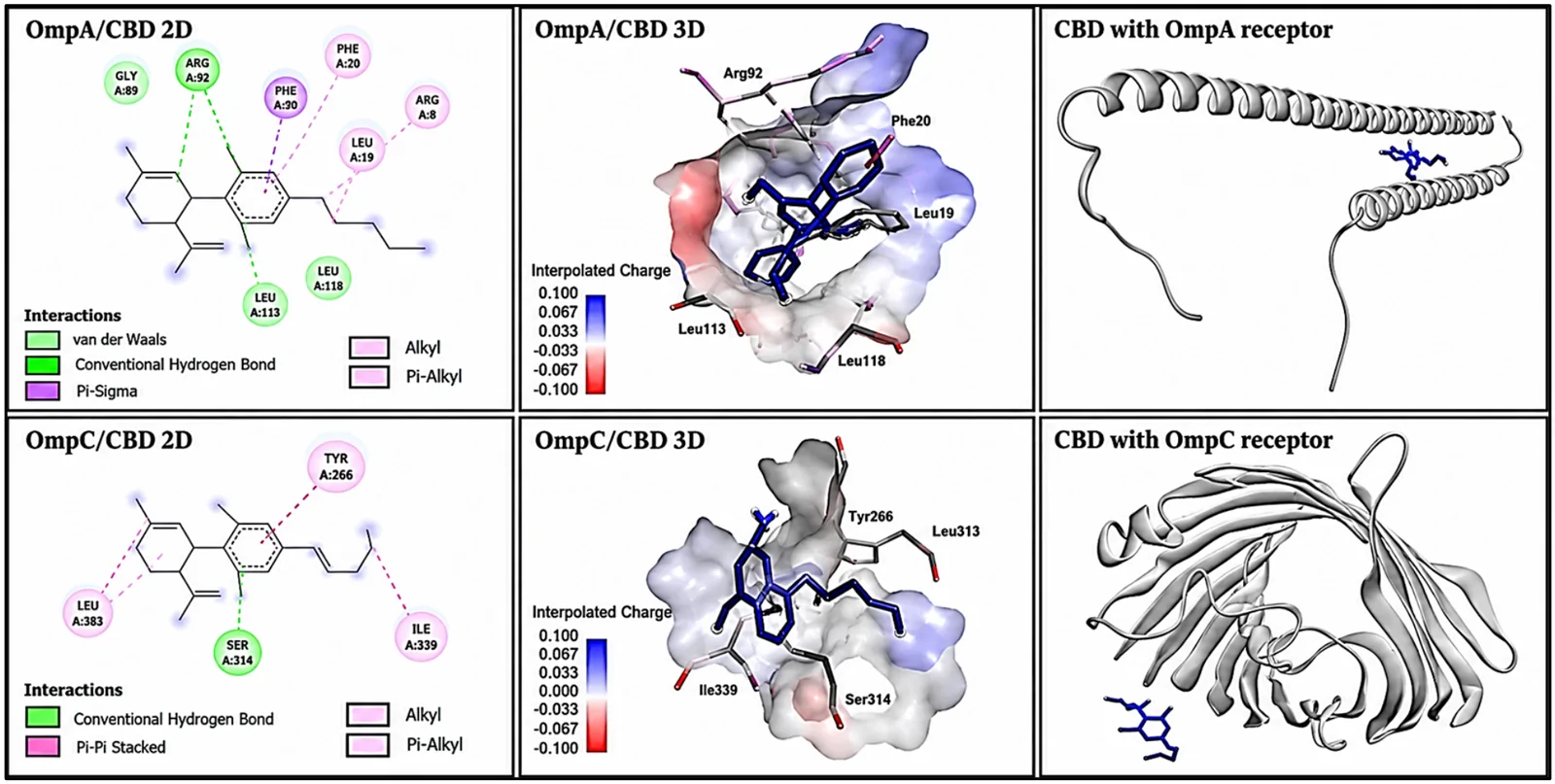
2D and 3D representations of predicted CBD interactions with OmpA and OmpC of *Salmonella* Typhimurium LT2. CBD is shown in blue in both the 2D and 3D representations. Panels were independently fitted for visualization and are not presented at a common scale.

The poses illustrated in Figure 9 include both 2D and 3D representations of CBD interactions with OmpA and OmpC, showing the predicted interacting residues and binding environments. The 2D pose showed that CBD interacts with various key residues in OmpA. Notable interactions include a conventional hydrogen bond with ARG120, a π-Sigma interaction with ARG116, and a π-Alkyl interaction with PHE115. An additional hydrophobic interaction with LEU123 was also observed. Furthermore, the 3D pose illustrates the spatial arrangement of CBD within the predicted binding region of OmpA. The interpolated charges visualized in the 3D pose show areas of positive potential (blue) and negative potential (red) around the interaction region. The protein backbone is shown in cartoon representation to provide an overview of the predicted binding environment.

The 2D pose of CBD with OmpC reveals interacting residues including TYR114, SER127, and ASN120, while the 3D illustration shows the positioning of CBD within the predicted OmpC binding region through hydrogen-bond and hydrophobic interactions. The 3D representation also shows the electrostatic potential around the predicted binding location, illustrating the charge distribution within the interaction region. Overall, Figure 9 identifies the predicted binding locations, interacting residues, and non-covalent contacts associated with CBD–OmpA and CBD–OmpC docking complexes.

**Figure 10:**
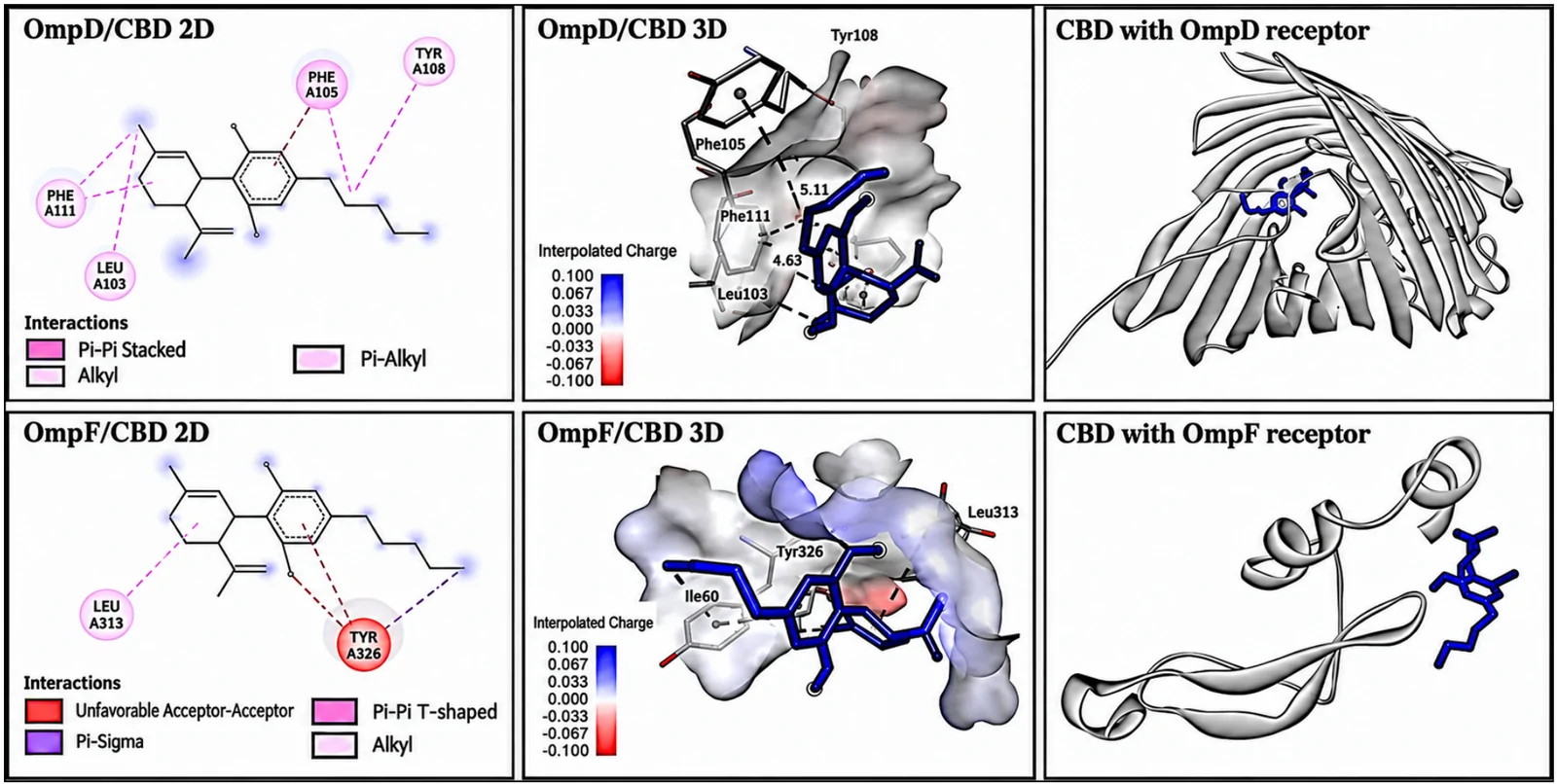
2D and 3D poses of *Salmonella* Typhimurium LT2 OmpD and OmpF interactions with CBD. The blue structures represent CBD in both the 2D and 3D conformations. Panels were independently fitted for visualization and are not presented at a common scale.

Figure 10 shows the predicted interactive poses for CBD with OmpD and OmpF of *Salmonella* Typhimurium LT2. The poses illustrated include both 2D and 3D representations, showing the predicted interacting residues and binding environments. The 2D pose showed that CBD interacts with various key residues in OmpD and OmpF. Notable interactions between CBD and OmpD include a π–π stacked interaction with PHE105, π-Alkyl interactions with TYR108 and PHE111, and an alkyl interaction with LEU103. The notable interactions between CBD and OmpF include a π–π T-shaped interaction with TYR265 and an alkyl interaction with LEU313. Furthermore, the 3D representations of OmpD and OmpF show the spatial arrangement of CBD within the predicted binding regions. For OmpD, residues including PHE105, TYR108, PHE111, and LEU103 are positioned around the predicted CBD-binding region. The interpolated charges are shown as areas of positive potential (blue) and negative potential (red). For OmpF, TYR265 and LEU313 are among the residues contributing to the predicted interaction with CBD. The 3D representation also shows the location of the predicted CBD-binding region relative to the rest of the OmpF structure.

**Figure 11:**
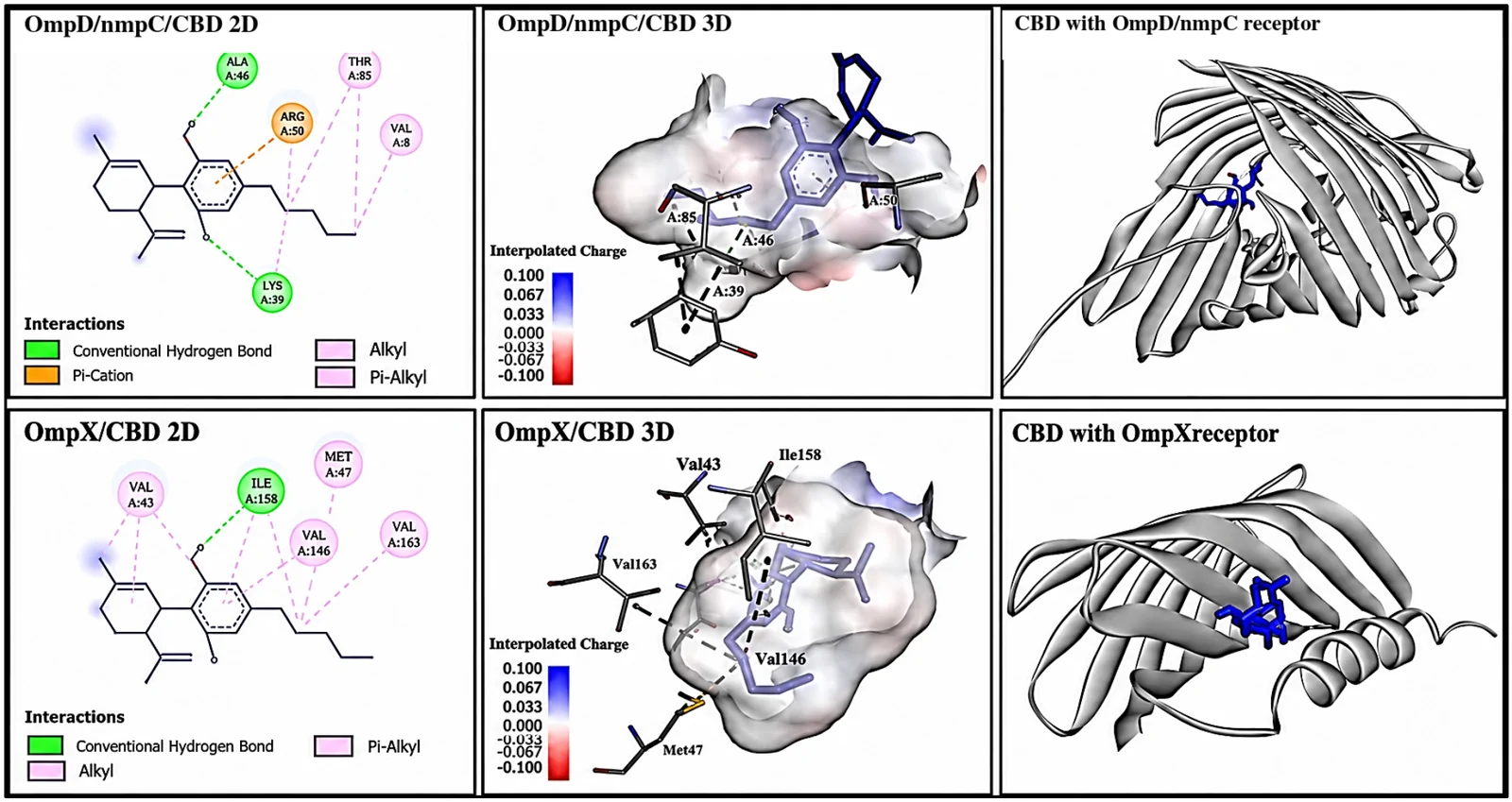
2D and 3D representations of predicted CBD interactions with OmpX and the OmpD/nmpC (legacy NmpC receptor entry) of *Salmonella* Typhimurium LT2. CBD is shown in blue in both the 2D and 3D representations. Panels were independently fitted for visualization and are not presented at a common scale.

Similar to Figure 9 and Figure 10, Figure 11 shows the predicted interactions of CBD with OmpX and the OmpD/nmpC (legacy NmpC receptor entry) of *Salmonella* Typhimurium LT2 in both 2D and 3D representations. The 2D pose of CBD with the OmpD/nmpC legacy receptor entry shows several interacting residues, including a conventional hydrogen bond with LYS37, a π-Cation interaction with ARG58, and π-Alkyl interactions with TYR35, ALA16, and VAL18. For OmpX, notable predicted interactions include a conventional hydrogen bond with ILE158 and π-Alkyl interactions with VAL43, VAL161, VAL163, and MET47. Furthermore, the 3D representations show the spatial arrangement of CBD within the predicted binding regions of both receptor entries. For the OmpD/nmpC legacy receptor entry, residues including LYS37, ARG58, ALA16, and VAL18 are positioned around the predicted CBD-binding region. For OmpX, ILE158, VAL43, VAL161, VAL163, and MET47 are among the residues associated with the predicted interaction. The charge distributions around the predicted binding regions are visualized as areas of positive potential (blue) and negative potential (red). The receptor configurations further show the location of CBD relative to the surrounding protein structures.

After assessing the 2D and 3D poses shown in Figure 9, Figure 10 and Figure 11, we further examined the predicted binding interactions based on interaction distance, interaction category, and interaction type. Table 1 provides a detailed overview of these parameters. The identified interactions included hydrogen bonds, hydrophobic interactions, and electrostatic interactions. The interaction distances generally ranged from approximately 2 to 5 Å. Hydrophobic interactions were predominant, particularly alkyl and π-Alkyl interactions.

### Outer Membrane Proteins of Different Salmonella enterica Strains at a 90% Residue-Identity Threshold

**Figure 12:**
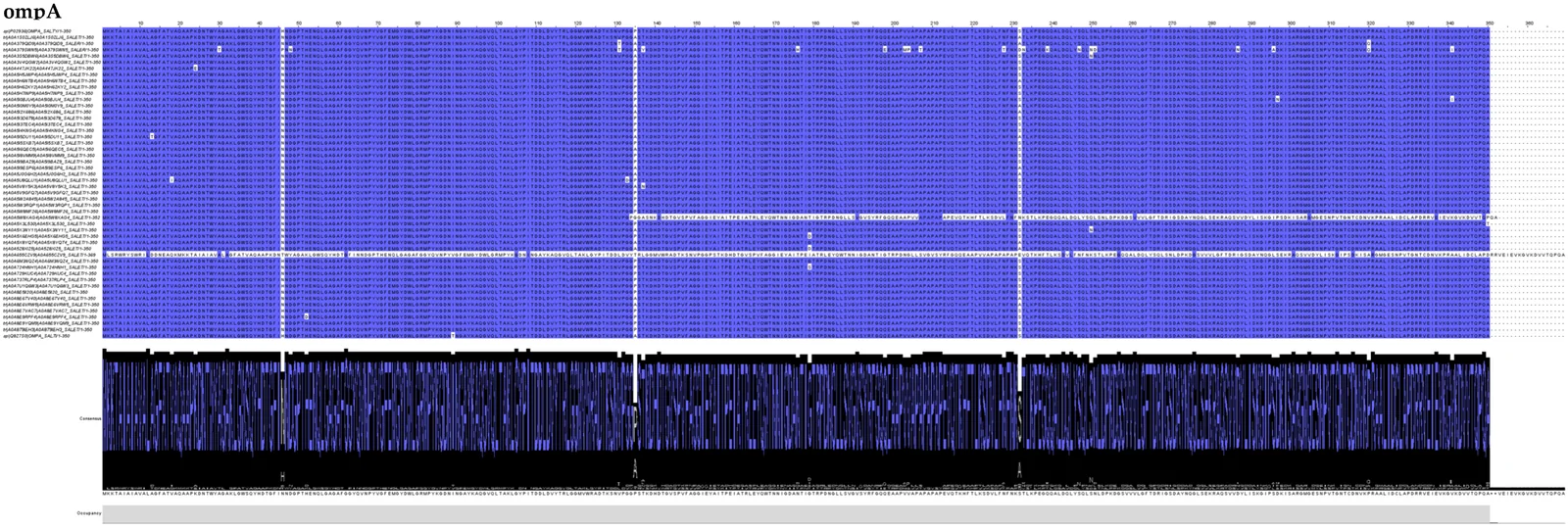

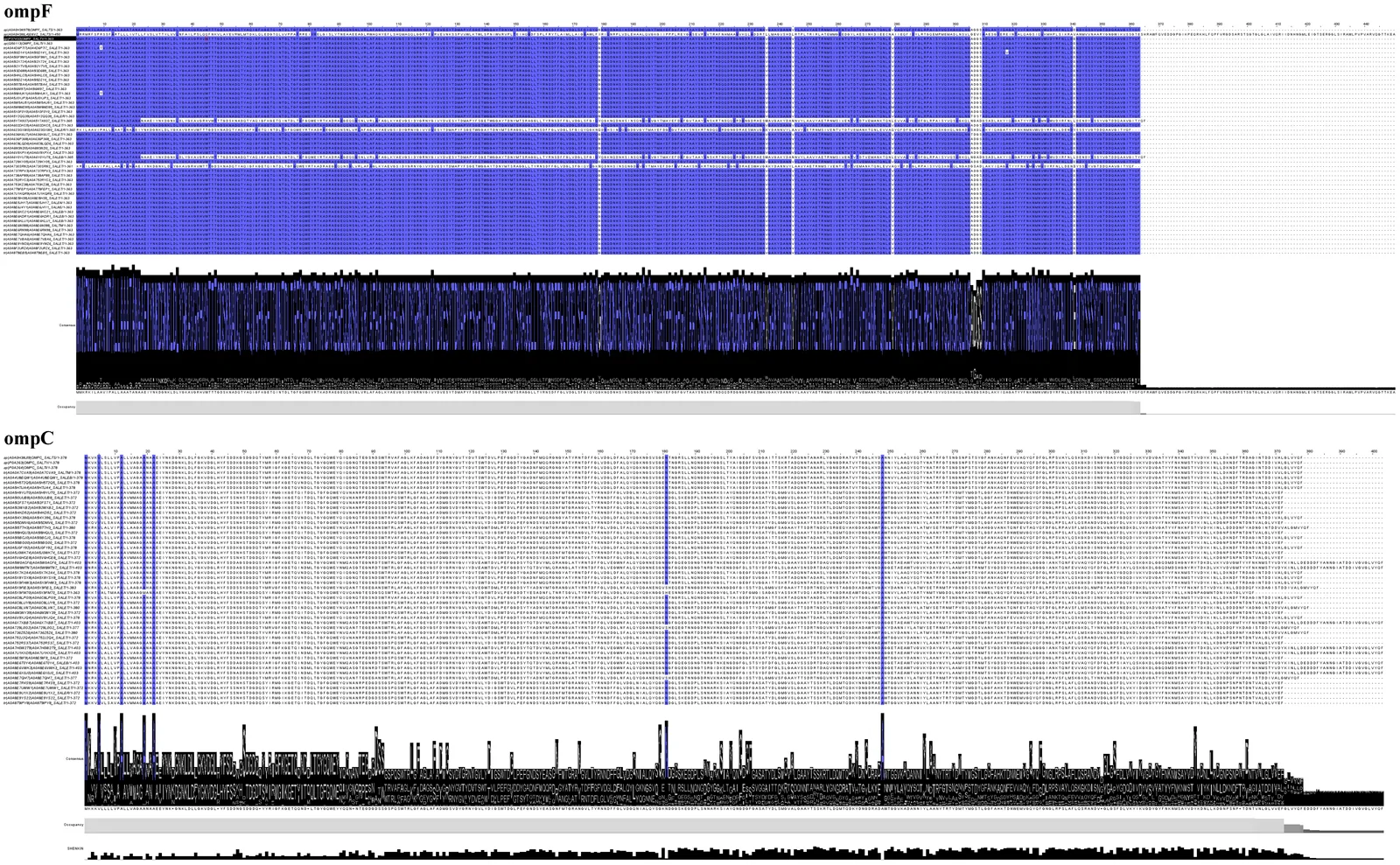

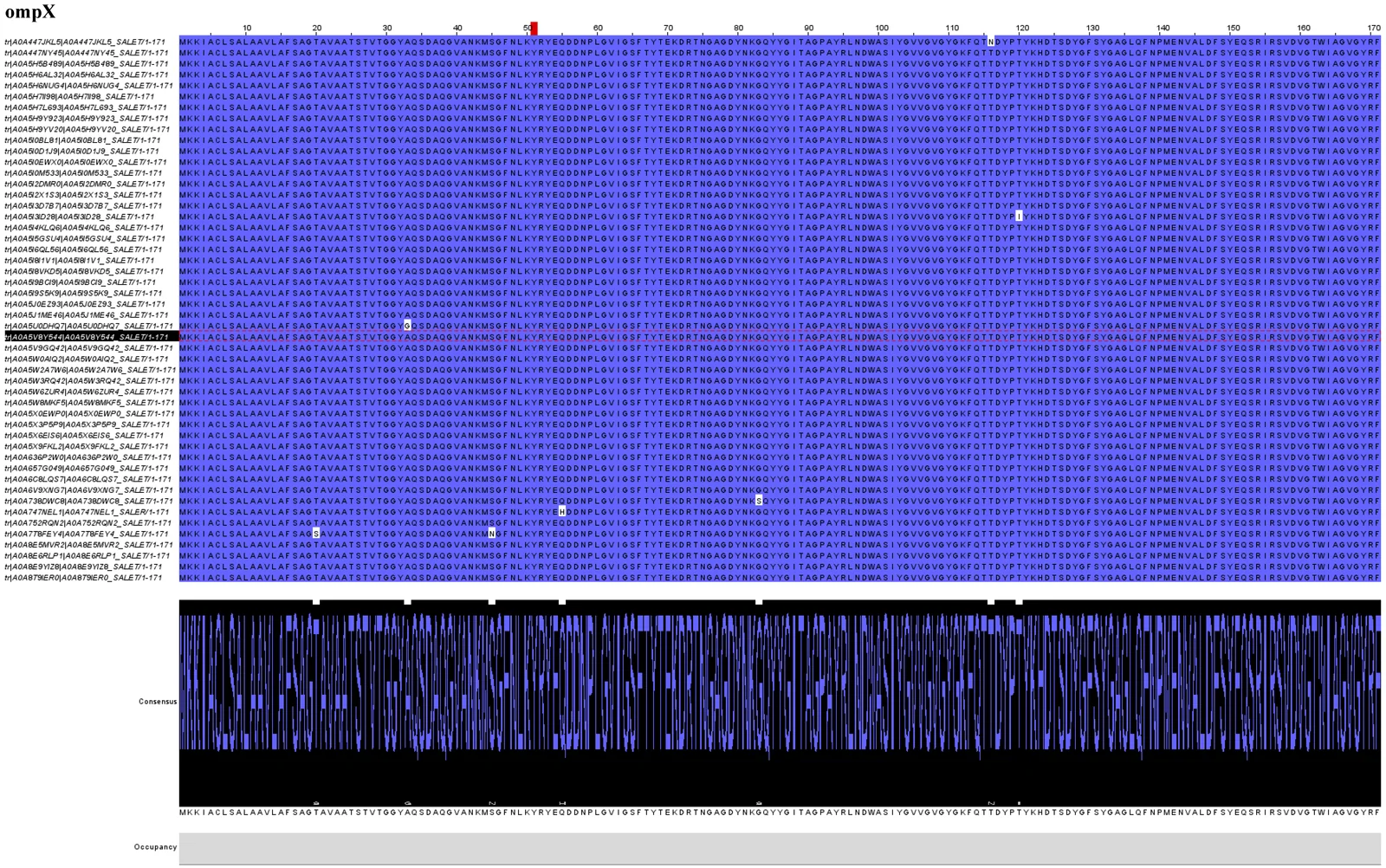

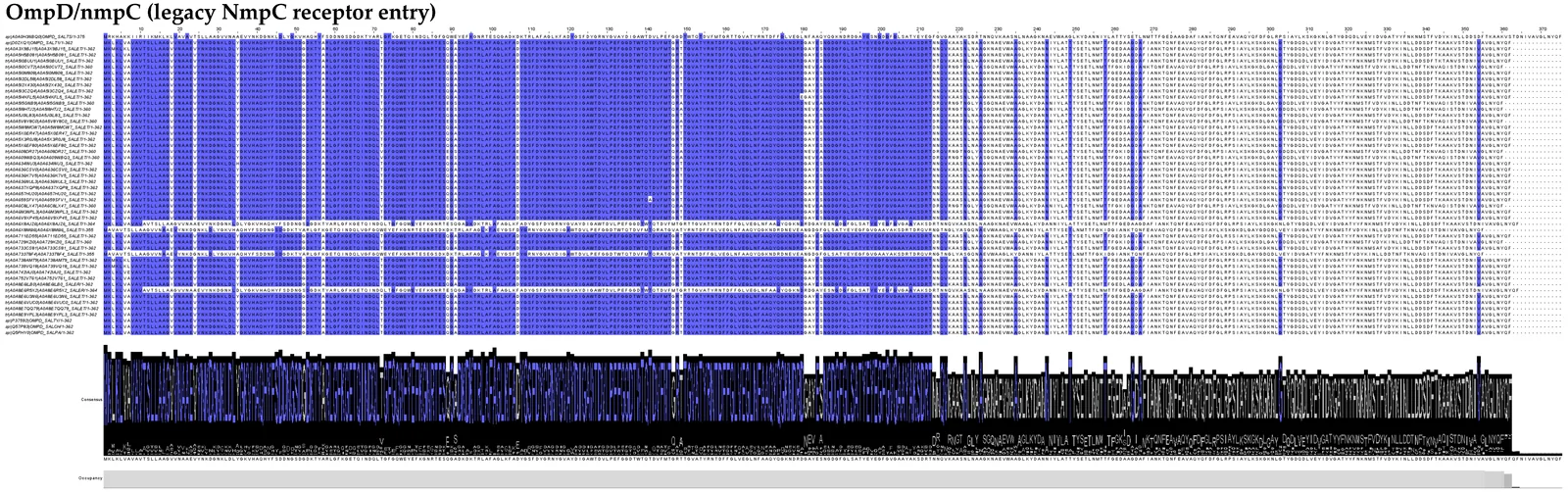
Overview of multiple-sequence alignments of outer membrane proteins from 50 *Salmonella enterica* strains, highlighting the broader pattern of sequence conservation at the 90% residue-identity threshold. The figure is intended to provide a global visualization of conserved and variable regions across the aligned sequences.

Furthermore, we carried out a comparative study of the outer membrane proteins (OMPs) extracted from 50 strains of *Salmonella enterica* with significant public health interest, as displayed in Figure 12. We achieved this by aligning the protein sequences and identifying highly conserved positions using a 90% residue-identity threshold. The conserved regions across the different strains are highlighted in Figure 12. The blue highlighted regions represent alignment positions meeting the 90% residue-identity threshold. These conserved regions may play important roles in maintaining the structure and function of the outer membrane proteins [15,16].

**Figure 13:**
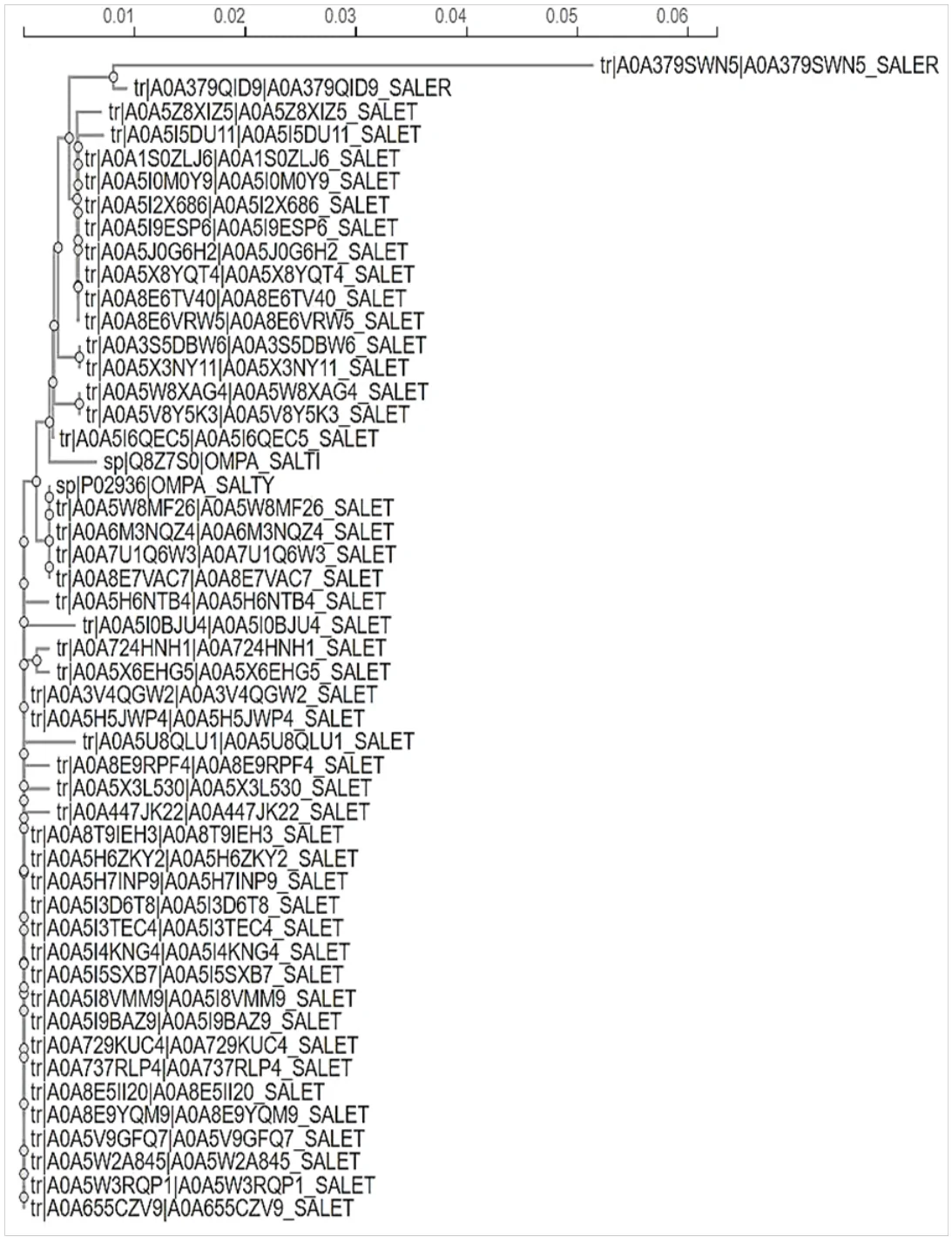
Phylogenetic tree showing the evolutionary relationships among OmpA sequences from 50 *Salmonella enterica* strains.

**Figure 14:**
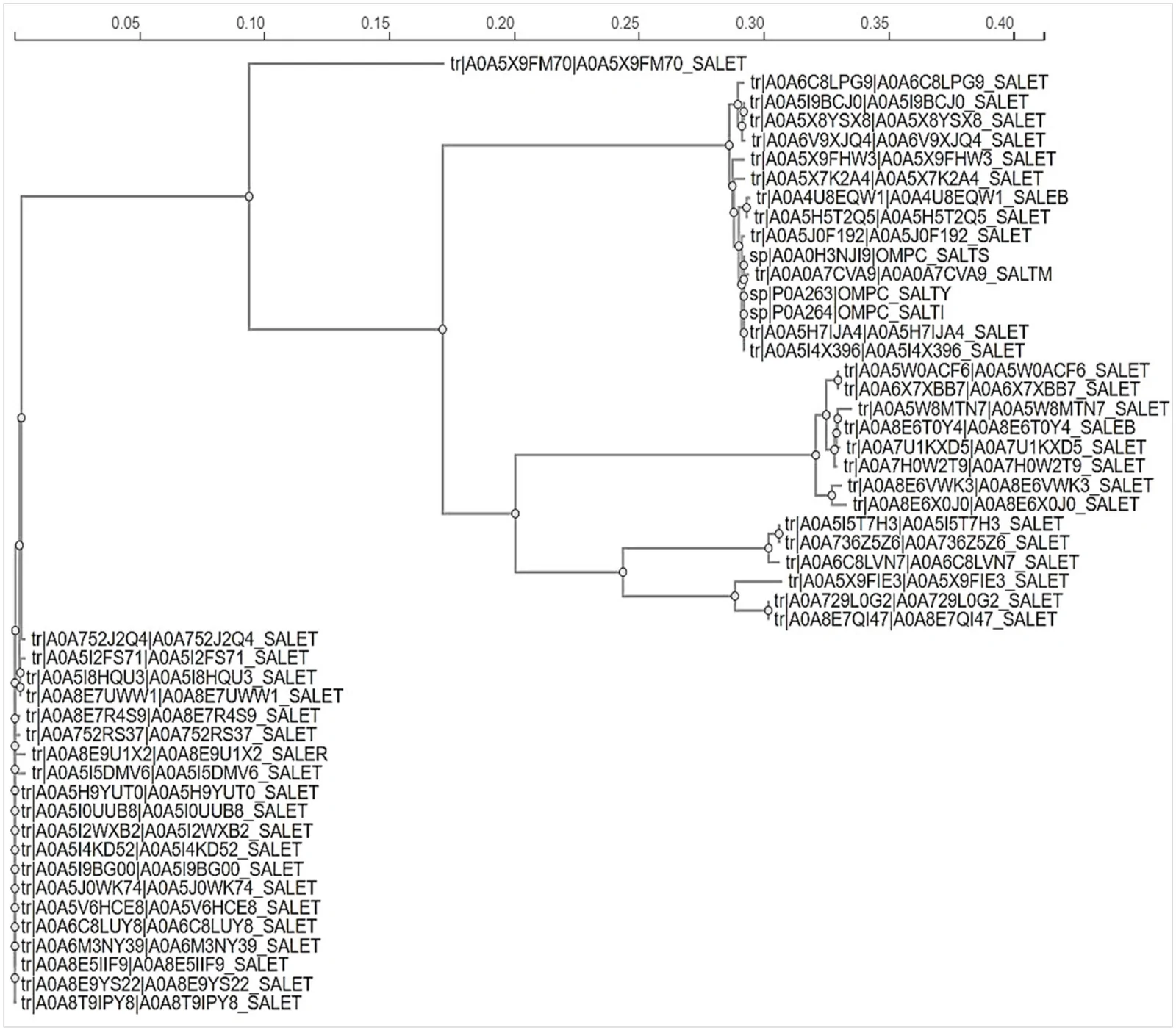
Phylogenetic tree showing the evolutionary relationships among OmpC sequences from 50 *Salmonella enterica* strains.

**Figure 15:**
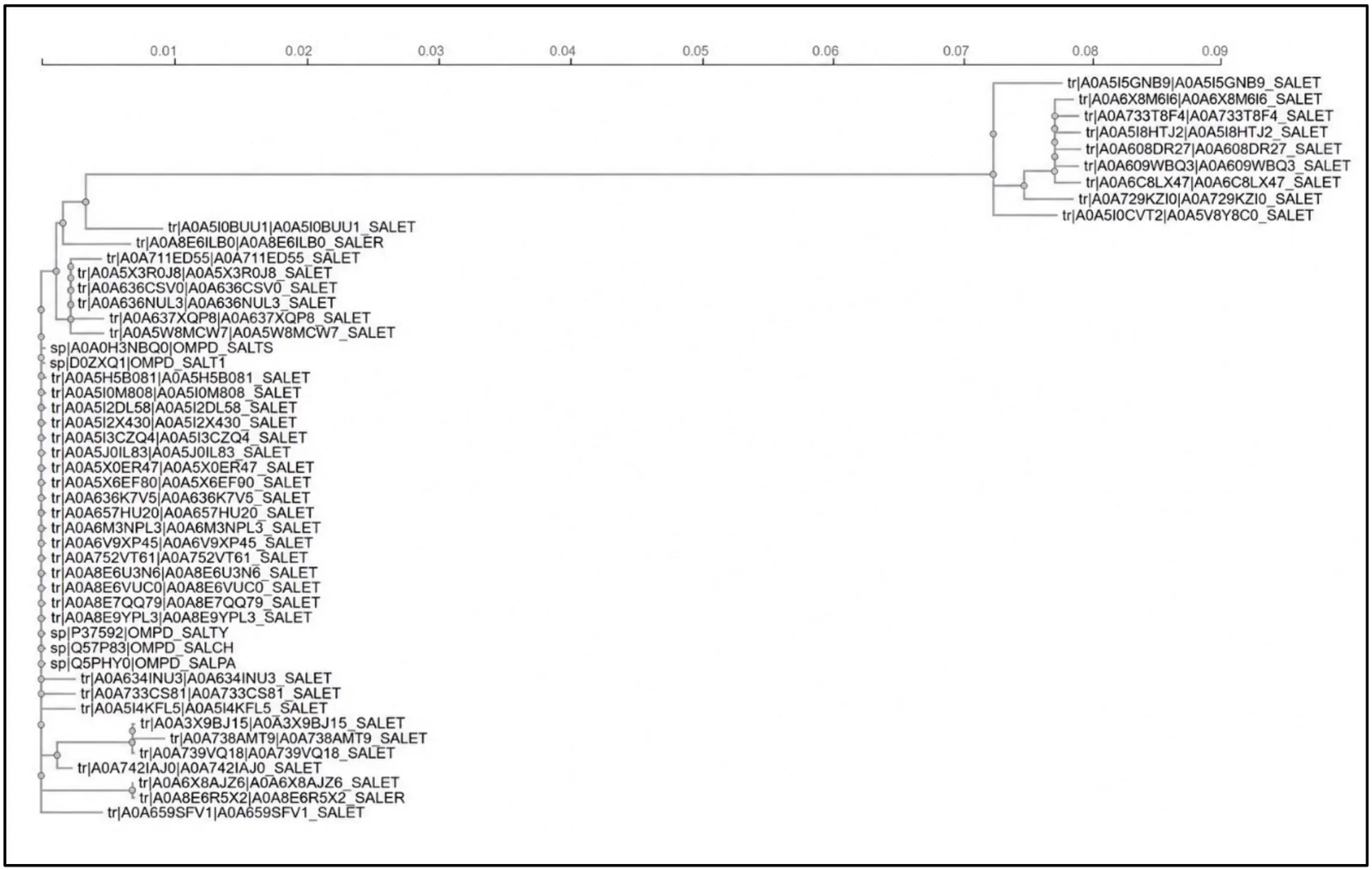
Phylogenetic tree showing the evolutionary relationships among OmpD sequences from 50 *Salmonella enterica* strains.

**Figure 16:**
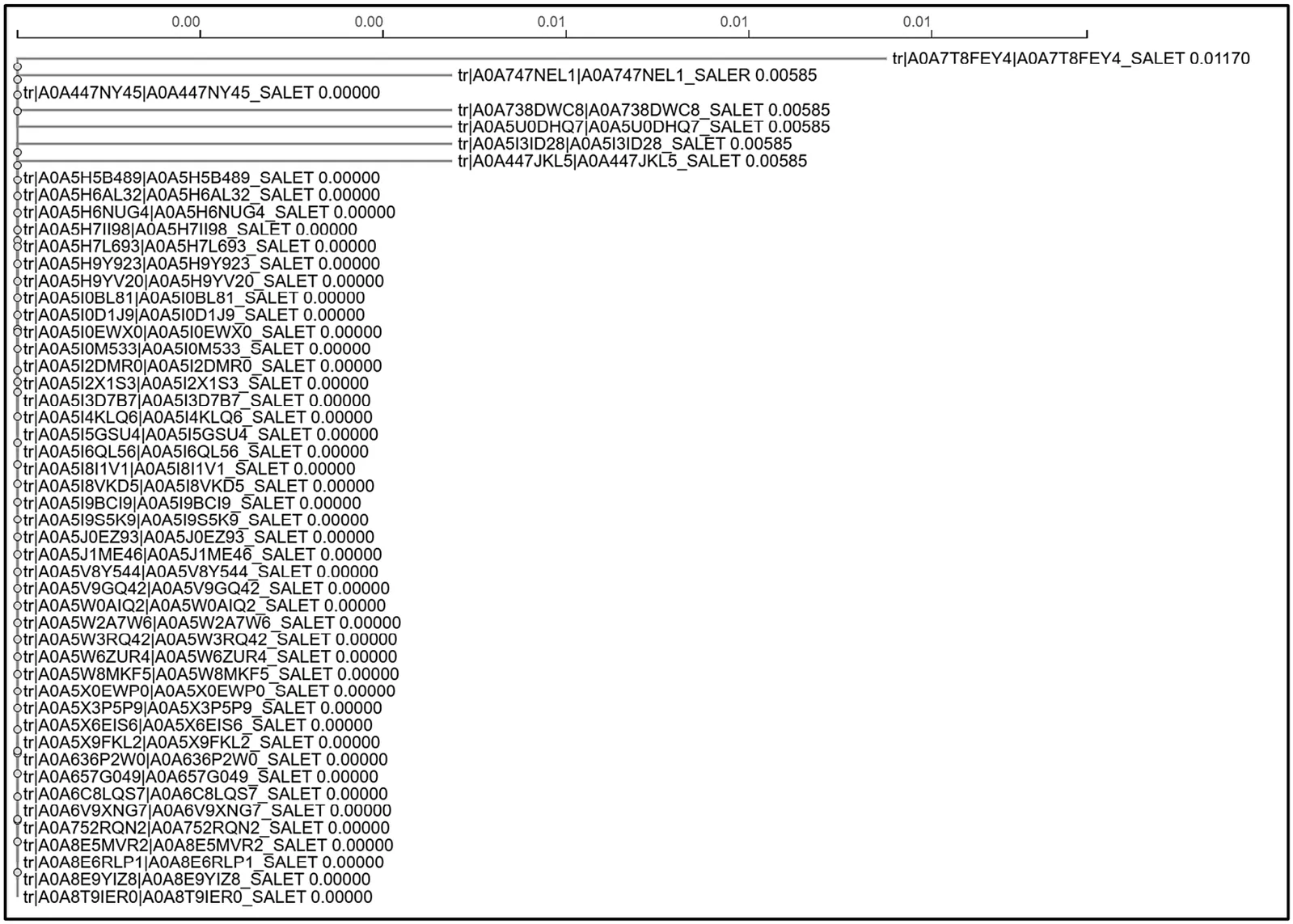
Phylogenetic tree showing the evolutionary relationships among OmpF sequences from 50 *Salmonella enterica* strains.

**Figure 17:**
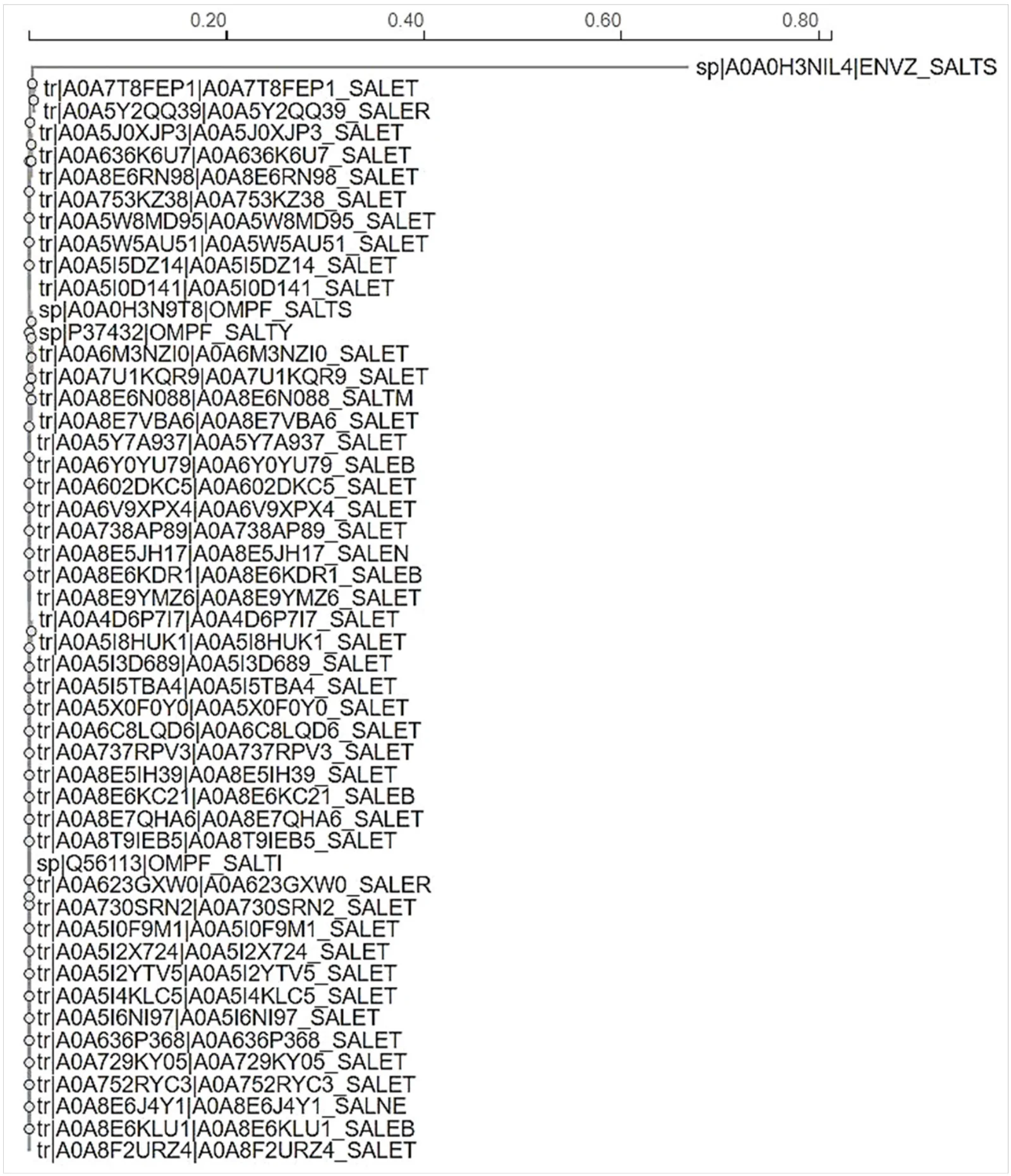
Phylogenetic tree showing the evolutionary relationships among OmpX sequences from 50 *Salmonella enterica* strains.

Phylogenetic analysis of OmpA, OmpC, OmpD, OmpF, and OmpX across the 50 *Salmonella enterica* strains revealed protein-specific clusters of closely related sequences, with branch lengths reflecting the relative sequence divergence within each alignment (Figure 13, Figure 14, Figure 15, Figure 16 and Figure 17). For OmpA, most entries grouped within closely related clusters, whereas PPQ29386.1OMPA_SALTY and Q8CZT5.1OMPA_SALTI appeared more divergent from many of the other OmpA sequences (Figure 13).

OmpC likewise formed several closely related clusters, while A0A6G6BLP9 and A0A6G8XCJ0 appeared more divergent from many of the other OmpC sequences (Figure 14).

OmpD sequences were generally closely related, although A0A5GNBJ0 and A0A5GN86L1 appeared more divergent from many of the other OmpD sequences (Figure 15).

OmpF sequences also formed closely related clusters, with A0A4747NEL1 and A0A378DWC8 appearing more divergent from many of the other OmpF sequences (Figure 16).

OmpX showed the same broad pattern of clustered related sequences; A0A7T8FEP1 and A0A3NIL4 appeared more divergent from many of the other OmpX sequences (Figure 17).

**Figure 18:**
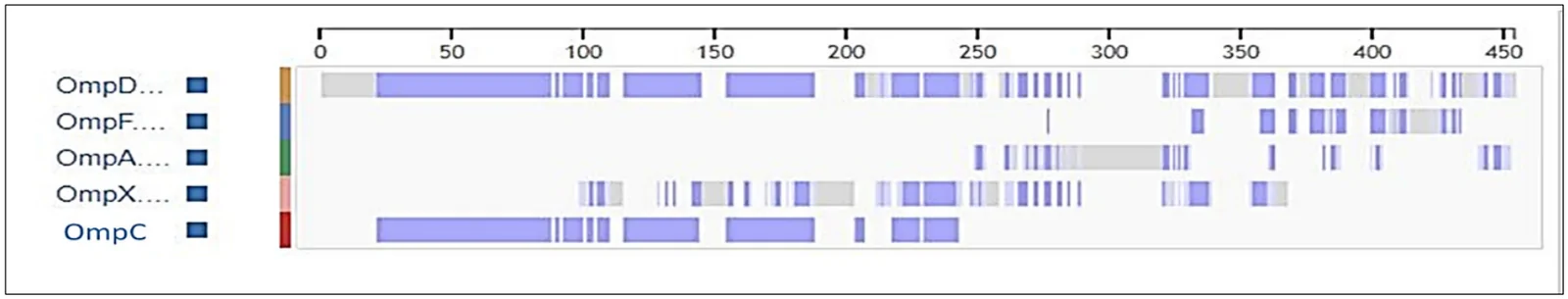
Structural alignment of OmpD, OmpF, OmpA, OmpX, and OmpC, highlighting their structural similarities and differences, with OmpD used as the reference structure.

Using OmpD as the reference structure, comparative structural alignment was performed for the evaluated *Salmonella* Typhimurium LT2 OMP structures, with the results summarized in Table 2 and Figure 18. OmpD contained 362 residues in the input structure, of which 346 were represented in the structural model; because OmpD served as the reference, comparative RMSD, TM-score, sequence identity, and aligned-residue values were not applicable. OmpF showed an RMSD of 3.72 Å, a TM-score of 0.11, 4% sequence identity within the structurally aligned region, and 41 aligned residues, with an input structural chain length of 65 residues. OmpA showed an RMSD of 5.12 Å, a TM-score of 0.11, 2% sequence identity, and 31 aligned residues, with a structural chain length of 136 residues. OmpX showed an RMSD of 5.55 Å, a TM-score of 0.24, 9% sequence identity, and 74 aligned residues, with a structural chain length of 171 residues. OmpC showed the greatest structural similarity to the OmpD reference among the compared structures, with an RMSD of 0.72 Å, a TM-score of 0.51, 72% sequence identity within the aligned region, and 177 aligned residues. The OmpC input structure contained 338 residues, of which 178 were represented in the structural comparison.

**Figure 19:**
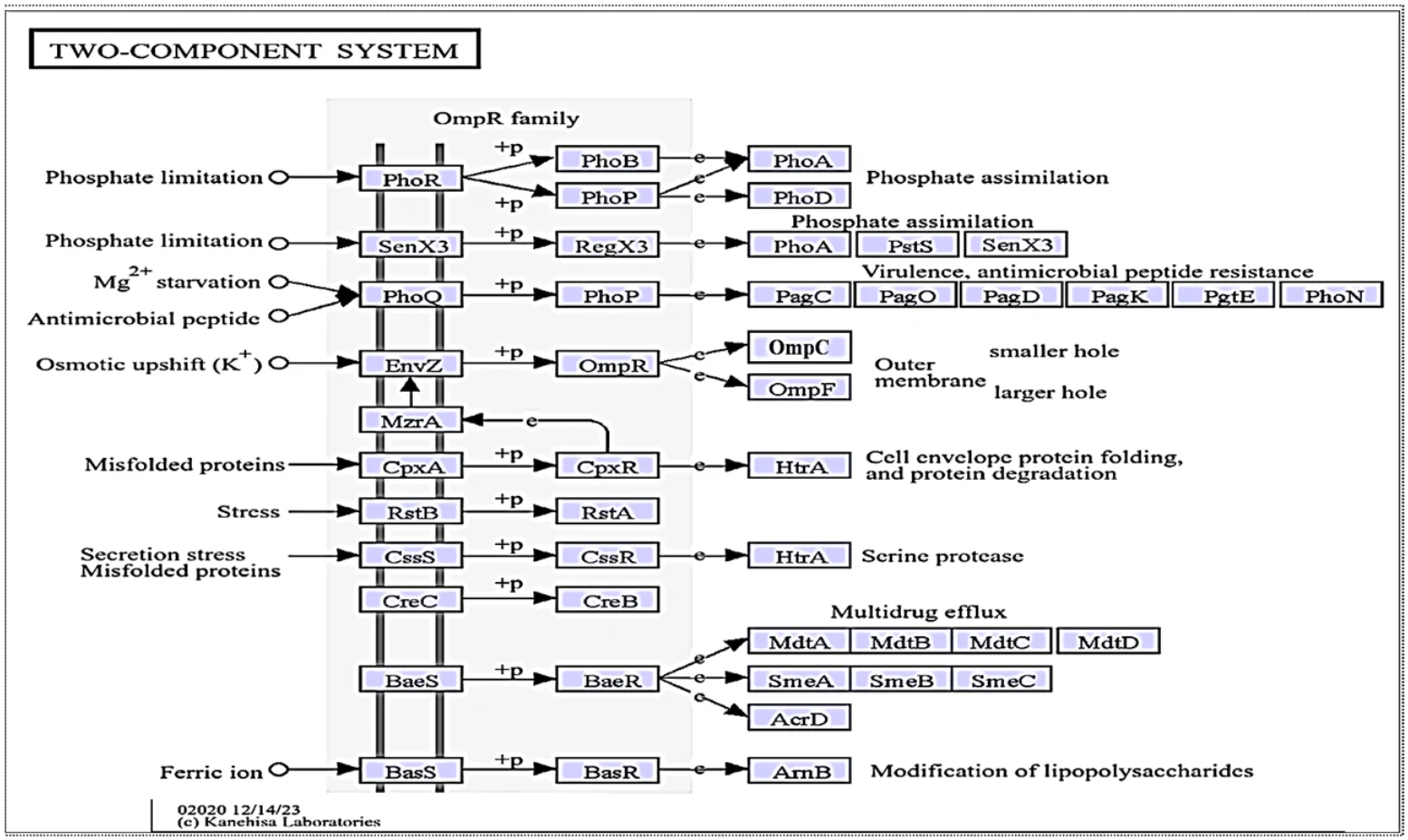
KEGG two-component-system pathway providing functional context for OmpC and OmpF in the response of *Salmonella* Typhimurium LT2 to environmental stimuli.

Figure 19 shows the KEGG functional context associated with OmpC and OmpF within the two-component system of *Salmonella* Typhimurium LT2. These porins participate in outer membrane permeability and are associated with regulatory responses to environmental conditions, including osmoregulatory processes. The pathway map also provides broader context for interconnected systems involved in nutrient acquisition, environmental adaptation, and antimicrobial stress responses. This pathway analysis was used solely to contextualize the known biological functions of OmpC and OmpF and does not demonstrate that CBD alters these pathways.

**Figure 20:**
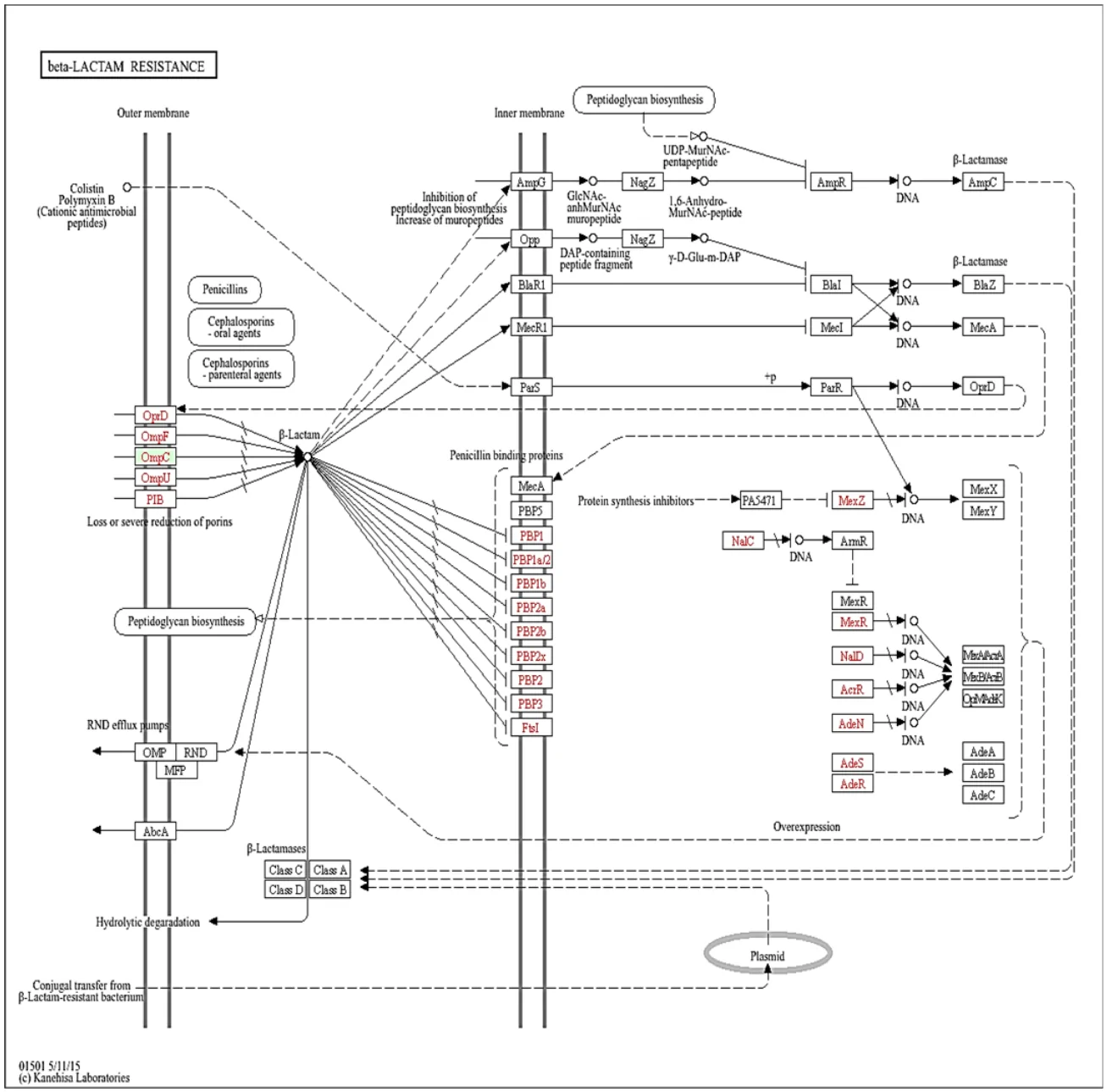
KEGG β-lactam-resistance pathway providing functional context for outer membrane porins, including OmpC and OmpF, in *Salmonella* Typhimurium LT2. Solid arrows indicate direct relationships; dashed arrows indicate indirect or multistep relationships; red labels mark highlighted components.

The KEGG β-lactam-resistance pathway was also examined to provide functional context for the roles of outer membrane porins in antimicrobial susceptibility, as shown in Figure 20. The pathway illustrates established resistance mechanisms involving altered outer membrane permeability, efflux systems, β-lactamases, penicillin-binding proteins, and regulation of peptidoglycan biosynthesis. OmpC and OmpF are relevant to this context because changes in porin abundance or function can influence antimicrobial entry. The pathway analysis itself did not evaluate the effects of CBD on β-lactam resistance or demonstrate that CBD modifies any of these resistance mechanisms.

ADMETLab 3.0 was used to predict selected physicochemical, drug-likeness, pharmacokinetic, transporter, metabolism, and toxicity-related properties of CBD, as summarized in Table 3. CBD satisfied the Lipinski rule-of-five criteria and had a QED score of 0.511. The predicted Caco-2 and MDCK permeability values were −4.746 and −4.582, respectively, while the PAMPA endpoint was classified as low permeability. CBD was predicted to interact with several transporters, including P-glycoprotein and BSEP, and was predicted to inhibit CYP2C9. The predicted plasma clearance (CLplasma) was 3.51, while the predicted half-life (T1/2) was 1.043. These values represent in silico predictions and require experimental pharmacokinetic and toxicity validation.

## 3. Discussion

Outer membrane proteins (OMPs) are integral to the pathogenicity of *Salmonella* Typhimurium LT2. They are also involved in nutrient transport, host immune interactions, and the structural integrity of *Salmonella* [1]. It is important to understand how CBD may interact with these proteins when exploring its potential as an antimicrobial agent against infections caused by *Salmonella* Typhimurium and other pathogenic organisms. Such understanding may provide valuable insights into CBD’s potential antibacterial mechanisms. In this study, we employed in silico analysis to examine the predicted interactions between CBD and various OMPs of *Salmonella* Typhimurium LT2. These interactions were characterized by hydrogen bonds, π-stacking interactions, and hydrophobic interactions. We retrieved the OMP structures in PDB format from the RCSB Protein Data Bank, and where experimentally determined structures were unavailable, amino acid sequences were retrieved from UniProtKB and modeled using SWISS-MODEL. Figure 1b presents the modeled structures of OmpX and OmpD together with their Ramachandran plots. Both OmpX and OmpD showed predominantly β-sheet conformations, with some regions corresponding to α-helical conformations. The 3D models showed transmembrane β-barrel structural features characteristic of outer membrane proteins [17,18]. The GMQE scores for OmpX and OmpD were 0.66 and 0.75, respectively. Figure 1a shows the 2D structure of CBD retrieved from PubChem. The hydroxyl groups, hydrocarbon chain, and aromatic rings of CBD provide chemical features capable of participating in hydrogen bonding, π-stacking, and hydrophobic interactions with protein residues [19,20,21]. The absence of MIC determination in the present study should therefore be considered in the context of our previous experimental work and other published studies demonstrating CBD antibacterial activity [10,11,12,13]. The present analysis was specifically designed to investigate potential molecular interactions underlying these previously observed antibacterial effects rather than to reproduce the susceptibility phenotype. Importantly, these previous MIC findings establish the antibacterial activity of CBD but do not independently validate the specific CBD–OMP interactions predicted in the present study.

CBD exhibited predicted interactions with each of the evaluated OMPs, although the docking scores and interaction profiles varied among the proteins. OmpA and CBD exhibited a predicted binding affinity of −5.9 kcal/mol (Figure 6 and Figure 7), with key interactions including a hydrogen bond at 3.2 Å and a hydrophobic π-Sigma interaction at 3.4 Å (Table 1; Figure 9). Hydrogen bonding and hydrophobic interactions can contribute to ligand accommodation within protein-binding regions. OmpA plays important roles in outer membrane stability and bacterial interactions with host cells [22,23,24]. Therefore, the predicted CBD–OmpA interaction identifies a potential site through which CBD may interact with this protein. However, these docking results do not demonstrate that CBD disrupts OmpA function, weakens the bacterial outer membrane, or alters bacterial adhesion or invasion; these possibilities require experimental validation.

CBD demonstrated a predicted binding affinity of −6.3 kcal/mol with OmpC (Figure 6 and Figure 7), with a hydrogen bond at 2.1 Å and a hydrophobic π–π stacked interaction at 4.4 Å among the identified contacts (Table 1; Figure 9). OmpC is an outer membrane porin involved in nutrient transport and membrane permeability [3]. The predicted positioning of CBD within the OmpC binding region therefore raises the possibility that CBD–OmpC interactions could influence porin-associated processes. Nevertheless, whether CBD-binding alters pore function, nutrient passage, membrane permeability, antimicrobial susceptibility, or host interactions cannot be determined from docking alone and requires direct experimental investigation.

CBD–OmpD interactions produced a predicted binding affinity of −6.2 kcal/mol (Figure 6 and Figure 7), with notable π–π stacked and hydrophobic alkyl interactions at 3.7 Å and 5.3 Å, respectively (Table 1; Figure 10). These interactions identify a computationally plausible CBD-binding region within OmpD. Given the involvement of OmpD in small-molecule transport and outer membrane-associated functions [25], the docking results provide a structural hypothesis for investigating whether CBD-binding affects these processes. However, effects on membrane permeability, environmental stress responses, immune evasion, or antimicrobial susceptibility cannot be inferred from the docking results alone.

For OmpF, CBD exhibited a predicted binding affinity of −5.7 kcal/mol (Figure 6 and Figure 7), with π-Sigma and π–π T-shaped interactions among the identified contacts (Table 1; Figure 10). OmpF functions as a porin that facilitates the passage of small molecules across the outer membrane and contributes to membrane permeability [3]. The predicted CBD–OmpF interaction therefore provides a structural basis for investigating whether CBD may influence OmpF-associated transport. However, the present computational findings do not establish changes in OmpF function, membrane permeability, nutrient transport, or susceptibility to antimicrobial agents.

Among the evaluated OMPs, OmpX exhibited the most favorable predicted CBD-binding affinity at −6.6 kcal/mol (Figure 6 and Figure 7), with several interactions, including a π-Sigma interaction at 3.5 Å and hydrophobic contacts (Table 1; Figure 11). Given the reported involvement of OmpX-family proteins in membrane-associated processes and host interactions [7], this predicted interaction identifies OmpX as a candidate for further mechanistic investigation. The docking results, however, do not demonstrate that CBD inhibits OmpX function or consequently alters membrane integrity, biofilm formation, bacterial persistence, virulence, immune evasion, or antimicrobial susceptibility.

The OmpD/nmpC (legacy NmpC receptor entry) also exhibited a favorable predicted interaction with CBD, with a binding affinity of −6.4 kcal/mol (Figure 6 and Figure 7) and hydrogen-bond and other non-covalent interactions within the predicted binding region (Table 1; Figure 11). These findings support a computationally plausible interaction between CBD and the OmpD/nmpC legacy receptor entry. As clarified above, this historical receptor entry is not interpreted as an additional independent OMP target. Any potential consequences for protein activity, bacterial survival, or virulence remain hypothetical and require experimental validation.

The alternative docking poses showed varying RMSD lower-bound and upper-bound values relative to the top-ranked poses (Figure 8). These RMSD values describe the geometric divergence of the alternative docking poses from the reference pose and should not be interpreted as evidence of time-dependent stability or functional disruption of the proteins. Collectively, the docking results presented in Figure 6, Figure 7, Figure 8, Figure 9, Figure 10 and Figure 11 and Table 1 identify plausible CBD-binding configurations and interacting residues within the evaluated OMPs. These findings provide testable structural hypotheses regarding possible CBD–OMP interactions but do not, by themselves, demonstrate functional inhibition or downstream effects on membrane integrity, bacterial survival, virulence, or antimicrobial activity.

We conducted phylogenetic analyses on outer membrane protein sequences from fifty different *Salmonella enterica* strains to examine the evolutionary relationships and sequence conservation of the OMPs considered in this study. The comparative sequence analysis identified highly conserved regions across the evaluated *Salmonella enterica* strains. Conserved regions may be important for maintaining protein structure and function and can therefore provide useful information when considering potential antimicrobial targets [26]. Additionally, examining the evolutionary relationships and divergence of OMP sequences among different *Salmonella enterica* strains provides insight into the degree to which these proteins are conserved or variable across the strains analyzed. In the context of the present study, conservation of OMP sequences may be relevant when considering whether predicted CBD-binding regions are shared among multiple *Salmonella* strains. However, sequence conservation alone does not demonstrate that CBD will exhibit broad-spectrum antibacterial activity or that targeting conserved regions will prevent the development of resistance [26].

The comparative sequence analysis of OMPs from fifty *Salmonella enterica* strains showed several highly conserved alignment positions based on the 90% residue-identity threshold used in this study. This threshold was applied to identify highly conserved positions within the multiple-sequence alignments and was not used to construct the phylogenetic trees. Figure 13, Figure 14, Figure 15, Figure 16 and Figure 17 illustrate the evolutionary relationships and sequence diversity of the evaluated OMPs. The trees showed distinct clusters of closely related sequences together with more divergent sequences among the different strains. These observations demonstrate varying degrees of sequence relatedness among the OMPs examined. Together, the conservation and phylogenetic analyses provide a basis for further investigating whether predicted CBD-binding regions are conserved across *Salmonella* strains; however, the present analysis does not establish CBD as a broad-spectrum antibiotic or demonstrate that conserved OMP regions are functionally inhibited by CBD.

Another dimension of this exploration was assessing the structural similarities and divergence of the outer membrane proteins to understand the structural conservation and diversity among these OMPs in *Salmonella enterica*. We used OmpD as the reference structure [18]. The 3D structural alignment showed both conserved and variable structural regions among the evaluated OMPs. The degree of structural similarity differed among the proteins, with OmpC showing the greatest structural similarity to the OmpD reference based on the comparative alignment metrics presented in Table 2 and Figure 18. These structural similarities and differences provide additional context for interpreting the predicted CBD–OMP interactions observed in the docking analyses. However, structural similarity alone does not demonstrate that CBD disrupts bacterial functions, reduces the development of antimicrobial resistance, or produces equivalent biological effects across the different OMPs. Rather, the structural comparison provides a basis for further experimental investigation of whether shared or distinct structural features influence CBD-binding and OMP function.

We used KEGG mapping to examine the functional and regulatory context associated with OmpC and OmpF. The PhoBR system is involved in the regulation of phosphate uptake and metabolism under phosphate-limiting conditions, providing broader context for nutrient acquisition and environmental adaptation in *Salmonella* [27]. The PhoP/PhoQ system responds to environmental signals, including changes in magnesium availability, and regulates genes involved in maintenance of the bacterial envelope, stress adaptation, and resistance to antimicrobial peptides [28]. Through these regulatory responses, alterations in lipopolysaccharide (LPS) and other outer membrane components may contribute to bacterial adaptation and interactions with host defenses. As porins, OmpC and OmpF facilitate the passive diffusion of small hydrophilic molecules across the outer membrane, with their relative expression influenced by environmental conditions, including osmolarity. Their regulation by the EnvZ/OmpR system contributes to the balance between membrane permeability and adaptation to changing environmental conditions, as illustrated in Figure 19. As shown in Figure 12, both OmpC (Figure 14) and OmpF (Figure 16) display substantial sequence conservation across the fifty different *Salmonella* strains examined. This conservation identifies these porins as potentially relevant targets for further investigation of CBD–OMP interactions across diverse Salmonella strains. However, sequence conservation and predicted binding alone do not demonstrate broad-spectrum antibacterial activity. Furthermore, conserved regions of bacterial proteins may represent useful targets for antimicrobial development because they are generally less variable than other regions of the proteins. The docking results showed predicted interactions of CBD with OmpC and OmpF; however, these findings do not establish that CBD alters the activity of regulatory systems such as PhoBR, PhoP/PhoQ, or EnvZ/OmpR. Any effects of CBD–porin interactions on bacterial stress adaptation, membrane permeability, or susceptibility to antimicrobial agents therefore require experimental validation. Figure 19 provides functional context for the involvement of OmpC and OmpF in broader outer membrane and LPS-associated regulatory processes relevant to bacterial adaptation and antimicrobial susceptibility [29,30]. Thus, the established roles of these porins in solute transport and membrane permeability provide a biological framework for investigating whether CBD–porin interactions influence compound uptake or intracellular accumulation; this possibility warrants further investigation.

Another essential pathway considered in the KEGG analysis was β-lactam resistance. Understanding the mechanisms by which *Salmonella* Typhimurium LT2 can reduce susceptibility to β-lactam antibiotics provides useful functional context for the development of antimicrobial strategies. β-Lactam antibiotics, including penicillins, carbapenems, and cephalosporins, primarily target bacterial cell-wall synthesis by interfering with enzymes involved in peptidoglycan assembly, which is essential for bacterial survival and cell-wall integrity [31]. Figure 20 illustrates the KEGG β-lactam-resistance pathway and the major cellular processes associated with this resistance phenotype. Outer membrane porins such as OmpC, OmpF, and OmpD can influence the passive diffusion of small hydrophilic molecules, including some antibiotics, across the bacterial outer membrane. Reduced porin expression or altered outer membrane permeability can therefore decrease the entry of β-lactam and other antimicrobial agents and limit their access to periplasmic or intracellular targets [32]. This reduction in drug uptake can contribute to decreased antimicrobial susceptibility.

Several enzymes also play important roles in peptidoglycan biosynthesis through the sequential synthesis and assembly of peptidoglycan precursors. MurA and MurB catalyze early consecutive steps in the formation of UDP-N-acetylmuramic acid, while MurC, MurD, MurE, and MurF participate in the formation of UDP-MurNAc-peptide intermediates. Lipid II is an essential peptidoglycan precursor, and its biosynthesis involves enzymes including phospho-N-acetylmuramoyl-pentapeptide-transferase (MraY) and MurG [33]. Penicillin-binding proteins (PBPs), located in the periplasm and associated with the bacterial inner membrane, participate in the final stages of peptidoglycan synthesis [34]. Their major functions include transglycosylation and/or transpeptidation reactions required for assembly and cross-linking of the bacterial cell wall. β-Lactam antibiotics inhibit susceptible PBPs, thereby interfering with peptidoglycan synthesis and potentially resulting in loss of cell-wall integrity and bacterial death. Resistance may arise through alterations in PBPs that reduce β-lactam binding or through acquisition of alternative low-affinity PBPs [35]. A well-characterized example is methicillin-resistant *Staphylococcus aureus* (MRSA), in which the *mecA* gene encodes the low-affinity PBP2a, allowing cell-wall synthesis to continue in the presence of many β-lactam antibiotics [36].

β-Lactamases, including enzymes belonging to TEM, SHV, and CTX-M families, can additionally hydrolyze the β-lactam ring and reduce antibiotic activity [37]. Genes encoding these and other β-lactamases may occur on mobile genetic elements, facilitating horizontal dissemination of resistance determinants. This is particularly relevant to extended-spectrum β-lactamases and other clinically important β-lactam-resistance determinants [36]. Efflux systems such as AcrAB-TolC, a well-characterized resistance-nodulation-division (RND) family system in Enterobacterales, can also contribute to multidrug resistance by exporting antimicrobial compounds and thereby reducing their intracellular concentrations [38]. In Figure 20, the pathway further provides context for regulatory networks associated with antimicrobial resistance, including regulators such as MarA, SoxS, and Rob. Collectively, these mechanisms illustrate the multifactorial nature of β-lactam resistance and provide a biological framework for understanding how membrane permeability, drug inactivation, target alteration, efflux, and gene regulation can contribute to antimicrobial susceptibility [37].

We included the β-lactam-resistance pathway in this study to illustrate established mechanisms of antimicrobial resistance and to provide functional context for the roles of outer membrane porins such as OmpC and OmpF. The KEGG analysis was not used to determine whether CBD alters the β-lactam-resistance pathway, and the pathway map should therefore not be interpreted as evidence of a direct effect of CBD on β-lactam resistance. The docking analysis independently identified predicted interactions between CBD and selected OMPs; however, these predictions alone do not demonstrate disruption of bacterial membranes, increased permeability to other antimicrobial agents, or enhancement of β-lactam activity. Such possibilities require direct experimental evaluation. This is distinct from β-lactam antibiotics, which exert their primary antibacterial effects by targeting enzymes involved in bacterial cell-wall synthesis.

CBD has also been investigated for antimicrobial activity against bacterial pathogens. Abichabki et al. reported an inability to readily select CBD resistance under the experimental conditions evaluated [11]. In contrast, previous experimental work from our group established a CBD-resistant *Salmonella* Typhimurium phenotype following prolonged exposure to CBD and investigated the structural and genetic changes associated with resistance development [39]. Comparative analysis of CBD-susceptible and CBD-resistant strains revealed differences in outer-membrane-associated components, including LPS and membrane sterol/fatty-acid profiles, indicating that remodeling of the bacterial envelope may accompany adaptation to CBD. The resistant phenotype was also associated with differential expression of several resistance- and membrane-associated genes, with prominent changes reported for *blaTEM*, *fimA*, *fimZ*, *int2*, *ompC*, and other regulatory genes [39]. Of particular relevance to the present study, the involvement of outer-membrane-associated changes and altered *ompC* expression suggested that OMPs may participate in the bacterial response to CBD. These previous experimental observations therefore provided additional biological rationale for the present investigation of predicted CBD interactions with *Salmonella* OMPs. However, the docking results presented here do not establish that binding to any individual OMP causes CBD susceptibility or resistance. Rather, they provide structural hypotheses that can be tested experimentally using CBD-susceptible and CBD-resistant strains.

We employed ADMETLab to predict various ADMET and drug-likeness parameters of CBD, considering absorption, distribution, metabolism, excretion, and toxicity, as elaborated in Table 3 [40,41,42,43,44]. We found that CBD possesses several properties potentially relevant to drug development, although there are also notable limitations and potential safety considerations. CBD has a molecular weight of 314.22 Da, a volume of 359.057, and a density of 0.875. It is moderately flexible, with a predicted flexibility value of 0.462. CBD has a Quantitative Estimate of Drug-likeness (QED) score of 0.511, indicating a mid-range predicted drug-likeness profile rather than a strongly favorable score. The predicted Caco-2 and MDCK permeability values were −4.746 and −4.582, respectively, while the PAMPA endpoint was classified as low permeability, indicating that permeability predictions differed among the evaluated in silico models. Previous studies have demonstrated that CBD bioavailability and pharmacokinetics can be influenced by formulation and route of administration [45,46,47,48]. Our group has also previously reviewed the potential application of carbon nanodots as a strategy for improving CBD delivery and therapeutic efficacy [49].

Additionally, CBD was predicted to inhibit key transporters, including P-glycoprotein (Pgp), MRP1, and BSEP [44]. These predicted transporter interactions may be relevant to CBD transport and disposition; however, their implications for CBD retention, antimicrobial efficacy, or resistance cannot be established from the present in silico analysis and require experimental validation. Potential transporter-mediated effects and drug–drug interactions therefore warrant further investigation. The ADMET analysis also predicted inhibition of CYP2C9, suggesting a potential for drug–drug interactions involving this metabolic pathway. In contrast, CBD was not predicted to inhibit CYP1A2, while predictions varied across the other CYP endpoints evaluated [44]. CBD’s predicted half-life was 1.043 h, while its predicted plasma clearance (CLplasma) was 3.51. These values should be interpreted as computational predictions rather than evidence of rapid in vivo elimination. Previous studies have demonstrated that CBD bioavailability and pharmacokinetics can be influenced by formulation and route of administration, further supporting continued investigation of alternative delivery strategies [45,46,47,48,49,50,51].

## 4. Materials and Methods

### 4.1. Protein Selection and Preparation

We initiated our exploration by selecting and preparing various outer membrane proteins that play crucial roles in the pathophysiology and pathogenicity of *Salmonella* Typhimurium LT2. The study included five independent OMP targets—OmpA, OmpC, OmpD, OmpF, and OmpX—together with the historical OmpD/nmpC receptor entry. These OMPs were chosen based on their contributions to bacterial cell-envelope structural integrity and other important functions, including roles relevant to antimicrobial susceptibility. The receptor originally designated “NmpC” in the docking dataset was re-examined during manuscript revision. Database verification showed that, in *Salmonella enterica* serovar Typhimurium LT2, *nmpC* is a synonym of *ompD* (locus tag STM1572; UniProt accession P37592; RefSeq accession NP_460531.1).

We used UniProtKB [https://www.uniprot.org/ (accessed on 1 September 2023)] and NCBI databases [https://www.ncbi.nlm.nih.gov/ (accessed on 1 September 2023)] to retrieve the sequences of these OMPs. UniProtKB and NCBI are comprehensive public resources for protein and nucleotide sequence information. The RCSB Protein Data Bank (PDB) [https://www.rcsb.org/ (accessed on 1 September 2023)] was used to retrieve available three-dimensional structures of the OMPs. In cases where the required 3D structures could not be retrieved from the PDB, we employed SWISS-MODEL [https://swissmodel.expasy.org/ (accessed on 1 September 2023)], a web-based homology-modeling tool, to generate structural models. AutoDockTools 4.2.6 was subsequently used to prepare the protein structures for molecular docking. Protein preparation included the addition of polar hydrogens, removal of water molecules, and addition of Kollman charges. The prepared structures were then used for the subsequent docking analyses.

### 4.2. Ligand Preparation

PubChem [https://pubchem.ncbi.nlm.nih.gov/compound/644019#section=2D-Structure (accessed on 1 September 2023)], a freely accessible chemical database managed by NCBI, was used to retrieve cannabidiol (CBD), our compound of interest. The CBD structure was prepared for molecular docking using PyMOL (open-source version 2.5.0) [https://github.com/schrodinger/pymol-open-source (accessed on 1 September 2023)]. The ligand structure was converted to PDB format and prepared in an appropriate form for the subsequent docking analysis.

### 4.3. Molecular Docking and Binding Affinity Calculations

AutoDockTools 4.2.6 was used for receptor preparation, and AutoDock Vina 1.2.5 was used to investigate the predicted interactions between CBD and the selected outer membrane proteins (OMPs). PyRx 1.1 served as the graphical interface for the Vina-based docking workflow. Each receptor was visually inspected using BIOVIA Discovery Studio Visualizer 2024 (Dassault Systèmes, San Diego, CA, USA), and the docking search-space center was selected separately to encompass the receptor region examined. No automated binding-pocket prediction algorithm was used. The receptor-specific AutoDock Vina center coordinates and search-space dimensions are presented in Table 4.

The search-space dimensions were 90 × 90 × 90 Å for OmpA, OmpC, OmpD, OmpX, and the historical OmpD/nmpC receptor entry and 40 × 40 × 40 Å for OmpF. The AutoDock Vina exhaustiveness parameter was set to 4 for all receptors, and a maximum of eight binding modes was generated per run. These broad search spaces were used for exploratory global docking across the selected receptor regions. Because the search spaces exceeded commonly recommended local-docking dimensions and exhaustiveness was set to 4, the results were interpreted as exploratory predictions rather than exhaustive identification of the global binding optimum. The generated poses were ranked according to their AutoDock Vina-predicted binding affinities. The pose with the most negative binding score was selected as the top-ranked configuration for subsequent protein–ligand interaction analysis and visualization. The RMSD lower-bound (RMSD l.b.) and upper-bound (RMSD u.b.) values describe the geometric divergence of each alternative pose relative to the top-ranked pose. These values were not interpreted as measures of time-dependent binding stability. Predicted binding affinities and interacting amino-acid residues were subsequently examined to characterize the computationally predicted CBD–OMP interactions. Because molecular docking provides model-dependent predictions, the resulting scores and poses were interpreted comparatively and not as direct experimental evidence of binding or functional inhibition.

PyRx incorporates the AutoDock Vina engine; therefore, PyRx–AutoDock Vina and standalone AutoDock Vina outputs were regarded as complementary implementations of the same Vina-based docking framework rather than as results from independent docking algorithms. AutoDockTools 4.2.6 was used for receptor preparation and grid configuration. Its grid-point and spacing parameters were configured independently and were not treated as equivalent to the search-space dimensions used by AutoDock Vina.

### 4.4. Interaction Analysis

BIOVIA Discovery Studio Visualizer 2024 (Dassault Systèmes, San Diego, CA, USA) was used to visualize the protein–ligand complexes corresponding to the top-ranked binding poses and to generate 2D and 3D interaction diagrams. These visualizations were used to identify the amino acid residues involved in the predicted CBD–OMP interactions and to characterize the types of non-covalent contacts within the predicted binding regions, including hydrogen-bond, hydrophobic, and electrostatic interactions. The 2D and 3D representations were used to illustrate ligand positioning and interaction geometry and were not interpreted as evidence of protein stability, time-dependent binding behavior, or CBD-induced conformational changes.

### 4.5. Phylogenetic and Sequence-Conservation Analysis

Protein sequences representing each OMP from 50 *Salmonella enterica* strains, including *Salmonella enterica* serovar Typhimurium LT2, were aligned separately using Clustal Omega [https://www.ebi.ac.uk/jdispatcher/msa/clustalo (accessed on 1 November 2023)]. The resulting multiple-sequence alignments were visualized in Jalview [https://www.jalview.org/ (accessed on 1 November 2023)] to examine residue conservation and sequence variation. A 90% residue-identity threshold was applied in Jalview to highlight highly conserved alignment positions. For each aligned position, this threshold represented the proportion of contributing sequences containing the consensus amino acid. Accordingly, a position was considered highly conserved when the same residue was present in at least 90% of the contributing sequences. For a position represented in all 50 sequences, this corresponded to the same residue occurring in at least 45 sequences.

The 90% cutoff was selected as a stringent descriptive criterion to emphasize highly conserved regions while minimizing the inclusion of moderately conserved positions. It was not treated as a statistical-significance threshold or a universal biological cutoff.

For each OMP, an unrooted neighbor-joining tree was generated from the corresponding Clustal Omega multiple-sequence alignment using EMBL-EBI Simple Phylogeny [https://www.ebi.ac.uk/jdispatcher/phylogeny/simple_phylogeny (accessed on 1 November 2023)]. Pairwise sequence distances were expressed as the proportion of amino-acid substitutions across aligned positions, excluding gaps. No distance correction or explicit amino-acid substitution model was applied. Bootstrap resampling and other branch-support analyses were not performed. Accordingly, the trees were interpreted as descriptive visualizations of sequence relatedness and divergence rather than as statistically supported phylogenetic reconstructions. Tree construction was independent of the 90% residue-identity threshold used to visualize conserved alignment positions.

### 4.6. Comparative Structural Analysis

For the comparative structural analysis of the OMPs, SWISS-MODEL and the RCSB PDB Pairwise Structure Alignment tool [https://www.rcsb.org/alignment (accessed on 1 December 2023)] were used. We aligned the protein structures to identify conserved and divergent structural regions. SWISS-MODEL was used to generate 3D structures for proteins whose experimentally determined structures were unavailable in the PDB. For these proteins, the corresponding amino acid sequences were retrieved from UniProtKB and NCBI databases and submitted to SWISS-MODEL for homology modeling. The most suitable predicted structural models were selected based on the available model-quality parameters and homologous template information.

Following prediction of the protein structures, the RCSB PDB Pairwise Structure Alignment tool was used to compare the various OMPs. This allowed the 3D structures of the different OMPs to be superimposed and compared. OmpD was used as the reference structure, and the other OMP structures were aligned against OmpD. The resulting alignments were used to evaluate structural similarities and differences among the proteins, including RMSD, TM-score, sequence identity within the aligned regions, and the number of aligned residues.

### 4.7. Pathway Mapping

For the pathway analysis, we focused on OmpC and OmpF because these porins were represented in KEGG pathway annotations relevant to the biological context examined in this study. Using the protein sequences retrieved from UniProtKB and NCBI databases, the sequences of OmpC and OmpF were submitted to BlastKOALA through the KEGG database [https://www.genome.jp/kegg/pathway.html (accessed on 1 December 2023)] to obtain functional annotations and corresponding KEGG Orthology (KO) assignments. The resulting KO information was then used to examine relevant KEGG pathways, including the two-component system and β-lactam-resistance pathway. Following pathway retrieval, the two-component-system map was examined with emphasis on components relevant to *Salmonella* Typhimurium LT2 and the functional context of OmpC and OmpF.

### 4.8. Data Analysis and Interpretation

Molecular docking was performed in three independent runs for each CBD–OMP complex using both the PyRx–AutoDock Vina workflow and standalone AutoDock Vina. For each independent docking run, the top-ranked pose based on predicted binding affinity was retained, and the corresponding binding scores from the three independent runs were used as replicate observations (n = 3). Binding-affinity values are reported as the mean of the three independent docking runs. Differences in mean predicted binding affinities among the evaluated OMP receptor entries were assessed using one-way analysis of variance (ANOVA), with *p* < 0.05 considered statistically significant. The eight poses generated within each individual docking run represented alternative predicted ligand conformations and were not treated as independent replicates. Statistical analyses and graphical presentations were performed using R 4.4.1 and Microsoft Excel 2016.

## 5. Conclusions

In this study, molecular docking and complementary in silico approaches were used to characterize predicted interactions between CBD and the *Salmonella* Typhimurium LT2 OMP dataset, comprising five independent targets—OmpA, OmpC, OmpD, OmpF, and OmpX—together with the historical OmpD/nmpC receptor entry. CBD exhibited favorable predicted binding across the evaluated receptor entries, with OmpX showing the most favorable predicted docking score among the independent OMP targets evaluated. The predicted complexes were supported by several non-covalent interactions, including hydrogen bonds, π-stacking interactions, and hydrophobic contacts. The historical receptor entry labeled “NmpC” also produced a favorable docking score; however, subsequent annotation verification established that nmpC is a synonym of ompD in *Salmonella* Typhimurium LT2.

Phylogenetic and structural analyses further demonstrated substantial conservation and structural diversity among the evaluated OMPs across the analyzed *Salmonella* strains, while pathway analysis provided biological context for the roles of OmpC and OmpF in membrane-associated processes. Collectively, these findings identify OMPs as plausible molecular interaction partners for CBD and provide structural hypotheses for investigating its antibacterial mechanisms. Importantly, molecular docking predicts binding propensity and interaction geometry but does not demonstrate functional inhibition. Therefore, potential effects of CBD–OMP interactions on membrane integrity, transport, bacterial survival, virulence, or antimicrobial susceptibility require confirmation through appropriate biochemical, biophysical, and microbiological experiments.

Employing KEGG pathway mapping, we further provided functional context for the established roles of OmpC and OmpF in membrane-associated regulatory and antimicrobial-resistance processes. These pathway associations are not direct evidence that CBD modulates the mapped pathways; rather, these pathways identify biological processes in which the predicted CBD–porin interactions may be investigated experimentally.

## Figures and Tables

**Table 1:** Predicted CBD-binding interactions with *Salmonella* Typhimurium LT2 outer membrane protein receptor entries.

**OmpA**				**OmpC**			
**Name**	**Distance**	**Category**	**Type**	**Name**	**Distance**	**Category**	**Type**
A:ARG120:N—:CBD:O	3.15	Hydrogen Bond	Conventional Hydrogen Bond	CBD::H—A:ALA129:O	2.12	Hydrogen Bond	Conventional Hydrogen Bond
A:ARG116:CG—:CBD:	3.35	Hydrophobic	π-Sigma	A:PHE88—:CBD:	4.40	Hydrophobic	π-π Stacked
A:ARG68—:CBD:	4.99	Hydrophobic	Alkyl	A:ALA129—:CBD:	4.86	Hydrophobic	Alkyl
A:ARG120—:CBD:	4.93	Hydrophobic	Alkyl	A:ALA129—:CBD::C	4.41	Hydrophobic	Alkyl
:CBD:—A:LEU69	4.65	Hydrophobic	Alkyl	A:PHE88—:CBD:	4.22	Hydrophobic	π-Alkyl
A:PHE115—:CBD:	5.00	Hydrophobic	π-Alkyl	A:TYR131—:CBD::C	5.31	Hydrophobic	π-Alkyl
				A:TYR149—:CBD:	4.69	Hydrophobic	π-Alkyl
				A:TYR149—:CBD::C	4.01	Hydrophobic	π-Alkyl
**OmpD**				**OmpF**			
**Name**	**Distance**	**Category**	**Type**	**Name**	**Distance**	**Category**	**Type**
A:PHE105—:CBD:	3.72	Hydrophobic	π-π Stacked	:CBD::C—A:TYR265	3.71	Hydrophobic	π-Sigma
:CBD::C—A:LEU103	5.30	Hydrophobic	Alkyl	A:TYR265—:CBD:	5.18	Hydrophobic	π-π T-shaped
A:PHE105—:CBD:	5.20	Hydrophobic	π-Alkyl	A:LEU313—:CBD:	5.51	Hydrophobic	Alkyl
A:TYR108—:CBD:	4.91	Hydrophobic	π-Alkyl				
A:PHE111—:CBD:	3.96	Hydrophobic	π-Alkyl				
A:PHE111—:CBD::C	4.63	Hydrophobic	π-Alkyl				
**OmpX**				**OmpD/nmpC (legacy NmpC receptor entry).**			
**Name**	**Distance**	**Category**	**Type**	**Name**	**Distance**	**Category**	**Type**
:CBD::C—A:PHE116	3.54	Hydrophobic	π-Sigma	A:LYS37:NZ—:CBD::O	3.22	Hydrogen Bond	Conventional Hydrogen Bond
A:ALA40—:CBD:	4.79	Hydrophobic	Alkyl	:CBD:H—A:ALA16:O	2.22	Hydrogen Bond	Conventional Hydrogen Bond
A:VAL163—:CBD:	5.12	Hydrophobic	Alkyl	A:ARG58:NH1—:CBD:	3.51	Electrostatic	π-Cation
:CBD::C—A:ARG159	4.00	Hydrophobic	Alkyl	A:LYS37—:CBD:	4.33	Hydrophobic	Alkyl
:CBD::C—A:VAL161	5.43	Hydrophobic	Alkyl	A:ARG58—:CBD:	4.41	Hydrophobic	Alkyl
:CBD:—A:MET47	5.15	Hydrophobic	Alkyl	:CBD::C—A:VAL18	4.30	Hydrophobic	Alkyl
:CBD:- A:ILE158	4.77	Hydrophobic	π-Alkyl	A:TYR35—:CBD:	5.21	Hydrophobic	π-Alkyl
:CBD:—A:VAL161	4.50	Hydrophobic	π-Alkyl	A:TYR35—:CBD::C	5.32	Hydrophobic	π-Alkyl

**Table 2:** Structural alignments of the various outer membrane proteins of *Salmonella* Typhimurium LT2.

Entry	Chain	RMSD	TM-Score	Identity	Aligned Residue	Sequence Length	Modeled Residue
OmpD.pdb	A	-	-	-	-	362	346
OmpF.pdb	A	3.72	0.11	4%	41	65	65
OmpA.pdb	A	5.12	0.11	2%	31	136	136
OmpX.pdb	A	5.55	0.24	9%	74	171	171
OmpC.pdb	A	0.72	0.51	72%	177	338	178

**Table 3:** ADMET Results for CBD.

Parameter	Score	Parameter	Score
Molecular Weight (MW)	314.22	CYP2C19 inhibitor	-
Volume	359.057	CYP2C19 substrate	+++
Density	0.875	CYP2C9 inhibitor	+++
Flexibility	0.462	CLplasma	3.51
Stereo Centers	2	T1/2	1.043
PAINS	0	Acute Aquatic Toxicity Rule	0
Lipinski Rule	Accepted	Genotoxic Carcinogenicity Mutagenicity Rule	0
QED	0.511	Non-Genotoxic Carcinogenicity Rule	0
SAscore	Easy	SureChEMBL Rule	0
Promiscuous compounds	0.114	FAF-Drugs4 Rule	1
Caco-2 Permeability	−4.746	Carcinogenicity	0.017
MDCK Permeability	−4.582	Eye Corrosion	0.004
PAMPA	---	Drug-induced Neurotoxicity	0.246
Pgp inhibitor	+++	hERG Blockers	0.539
Pgp substrate	--	hERG Blockers (10 um)	0.882
OATP1B1 inhibitor	++	NR-AhR	-
OATP1B3 inhibitor	-	NR-AR	---
BCRP inhibitor	+	NR-AR-LBD	---
MRP1 inhibitor	++	NR-Aromatase	++
BSEP inhibitor	+++	NR-ER	-
CYP1A2 inhibitor	---	Hematotoxicity	0.448
CYP1A2 substrate	---	NonBiodegradable	1
BBB	+++	AMES Toxicity	0.386

For the classification endpoints, the prediction probability values are transformed into six symbols: 0–0.1 (---), 0.1–0.3 (--), 0.3–0.5 (-), 0.5–0.7 (+), 0.7–0.9 (++), and 0.9–1.0 (+++).

**Table 4:** Receptor-specific AutoDock Vina parameters used for CBD docking.

Receptor	Center X (Å)	Center Y (Å)	Center Z (Å)	Search-Space Dimensions (Å)	Exhaustiveness	Maximum Modes
OmpA	5.049	−4.736	−3.288	90 × 90 × 90	4	8
OmpC	52.080	−6.408	−13.555	90 × 90 × 90	4	8
OmpD	−14.238	4.599	1.756	90 × 90 × 90	4	8
OmpF	31.214	33.839	73.061	40 × 40 × 40	4	8
OmpX	3.281	−5.938	−14.092	90 × 90 × 90	4	8
OmpD/nmpC (legacy NmpC receptor entry) *	−12.238	4.599	2.756	90 × 90 × 90	4	8

* OmpD/nmpC denotes the legacy receptor entry originally labeled NmpC in the original docking dataset; subsequent annotation verification identified *nmpC* as a synonym of *ompD* in *Salmonella enterica* serovar Typhimurium LT2.

## Data Availability

The data presented in this study are openly available in Preprints.org at 10.21203/rs.3.rs-4858257/v1.
